# Effects of different exercise modalities on blood pressure and endothelial function in prehypertension individuals: a systematic review and network meta-analysis

**DOI:** 10.3389/fcvm.2025.1550435

**Published:** 2025-06-25

**Authors:** Yingtian Yang, Qianyu Lv, Xinzheng Hou, Yanfei Lv, Xirui Zhang, Qian Wu, Xuejiao Ye, Chenyan Yang, Mingyu Huang, Shihan Wang

**Affiliations:** ^1^ Department of Cardiovascular Internal Medicine, Guang’anmen Hospital, China Academy of Chinese Medical Sciences, Beijing, China; ^2^ School of Management, Fudan University, Shanghai, China; ^3^ Department of Geriatrics, Baogang Hospital of Inner Mongolia, Baotou, China

**Keywords:** exercise, prehypertension, blood pressure, endothelial function, network meta-analysis

## Abstract

**Objective:**

To evaluate the relative impacts of various exercise protocols on blood pressure (BP) and endothelial function in prehypertension individuals.

**Methods:**

In this systematic review and network meta-analysis (NMA), PubMed, Cochrane Library, Web of Science, Embase, CINAHL, SPORTDiscus, and Rehabilitation & Sports Medicine databases were searched until September 12, 2024. Randomized controlled trials that examined the effectiveness of exercise treatments on people with prehypertension compared to a non-exercise control group or other exercise modes were included. A Bayesian NMA were applied to examine SBP, DBP, flow-mediated dilation (FMD), and pulse wave velocity (PWV). The surface underneath the cumulative ranking curve data was utilized to rate interventions. Confidence was evaluated using the CINeMA framework.

**Results:**

A total of 18 articles with 2,592 individuals were included in the NMA. Tai Chi demonstrated the greatest decrease in SBP of −8.67 mm Hg [95% credible interval (CI): −17.29 to −0.05], while isometric exercise training (IET) exhibited the most substantial enhancement in DBP of −4.61 mm Hg (95% CI: −8.11 to −1.11) and PWV of −0.82 m/s (95% CI: −1.58 to −0.06). Moderate-intensity continuous training yielded the largest improvement in FMD at 5.82% (95% CI: 5.41–6.22).

**Conclusion:**

Diverse exercise modalities can enhance BP and vascular function in prehypertensive patients. Overall, Tai Chi and IET are the most productive exercises for reducing BP, with IET being particularly effective in diminishing arterial stiffness. MICT stands out in enhancing endothelial function. Nevertheless, further comprehensive studies encompassing more diverse exercise treatments are necessary to ascertain the best exercise patterns and dosing strategies.

**Systematic Review Registration:**

https://www.crd.york.ac.uk/PROSPERO/display_record.php?RecordID=614765, identifier: CRD42024614765.

## 1 Introduction

Hypertension (HTN) represents a key risk factor for cardiovascular events, causing damage to multiple organs (1). Currently, the primary management of HTN remains long-term pharmacological treatment, which is associated with poor adherence, high long-term economic burden, and potential drug-related adverse effects (2). Therefore, advancing the frontline of HTN prevention and control is particularly crucial for disease prevention and prognosis improvement (3). The 7th Report of the Joint National Committee on Prevention, Detection, Evaluation, and Treatment of High Blood Pressure (JNC7) in 2003 first proposed the concept of prehypertension (PHT), characterized by systolic blood pressure (SBP) of 120–139 mm Hg and/or diastolic blood pressure (DBP) of 80–89 mm Hg (4). The 2018 China Hypertension Survey reported (5) that approximately 41.3% of adults were in a PHT state, with an estimated national PHT population of 435 million. As a transitional phase between normative blood pressure (BP) and clinical HTN, PHT is more likely to progress to HTN and cardiovascular diseases than those with normal BP (6, 7).

Endothelial dysfunction and vascular remodeling are critical characteristics of HTN and preliminary indications of atherosclerosis (8). Retrospective research of 1375 HTN individuals indicated that flow-mediated dilation (FMD) and pulse wave velocity (PWV) are independent risk factors for predicting the occurrence of coronary heart disease in hypertensive populations (9). FMD, a non-intrusive measurement of endothelial function, is regarded as the gold standard for evaluating vascular endothelial function in clinical research. FMD is an independent predictor of cardiovascular outcomes and can be utilized to identify subjects at increased risk for future cardiovascular events (10). Simultaneously, diminished FMD is significantly linked to the onset of HTN (11). PWV is an essential parameter to evaluate arterial stiffness. PWV levels over 10 m/s can forecast detrimental hemodynamic alterations, independently increasing cardiovascular risk (12, 13). Additionally, PWV may serve as a reliable marker for detecting target organ damage in patients with PHT (14). Consequently, efficient monitoring of vascular function in PHT patients offers a thorough insight into vascular health and the related risks of HTN. This is crucial for identifying high-risk individuals early, evaluating therapeutic outcomes, and formulating personalized treatment strategies.

Increasing evidence suggests that exercise interventions can effectively lower blood pressure in HTN and PHT patients (15, 16). Relevant guidelines also prioritize exercise as a management and treatment strategy for PHT people. This is because exercise not only offers potential cost-effectiveness compared to antihypertensive medications but also modifies the lifestyle of PHT patients, providing lifelong benefits (17, 18). A previous network meta-analysis on effective antihypertensive strategies for PHT patients confirmed that exercise is the most effective intervention (19). Moreover, recent research has demonstrated that exercise can significantly improve endothelial function in HTN and PHT patients, including enhancing FMD and reducing PWV (20, 21). Despite these benefits, there remains a lack of comprehensive evaluations assessing the impact of various exercise modalities on PHT individuals, particularly for improving vascular function indicators like FMD and PWV.

Network meta-analysis (NMA) expands upon pairwise meta-analysis (22), utilizing direct and indirect evidence to improve effect estimation and treatment ranking, even when specific interventions have never been compared in randomized controlled trials (RCTs) (23). This study aims to assess the effectiveness of various exercise protocols on BP and endothelial function in adults with PHT based on a comprehensive systematic review and network meta-analysis. Evidence-based guidance will help refine treatment strategies for PHT and optimize clinical management.

## 2 Methods

### 2.1 Study design and registration

Our review was conducted in alignment with the Cochrane Collaboration Handbook (24) and adhered to the Preferred Reporting Items for Systematic Reviews and Meta-Analyses (PRISMA) (25) as well as the extension statement for network meta-analyses (PRISMA-NMA) (26) reporting guidelines (Supplementary Table S1). *A priori* protocol has been registered in PROSPERO (registration number: CRD42024614765).

### 2.2 Search strategy

A complete search was conducted from PubMed (MEDLINE), Cochrane Library, Web of Science, Embase, CINAHL, SPORTDiscus, and Rehabilitation & Sports Medicine Source databases from inception until September 12, 2024. With the assistance of a research librarian proficient in scientific databases, we executed a search strategy utilizing a combination of medical subject heading (MeSH) terms and keywords pertinent to exercise, physical activity, prehypertension, and randomized controlled trials. Manual searches of the reference lists from prior and analogous systematic reviews and meta-analyses were conducted to uncover additional papers not found in the initial search. The comprehensive search procedures are outlined in Supplementary Table S2.

### 2.3 Inclusion and exclusion criteria

Eligibility criteria were developed according to the PICOS framework (Population, Intervention, Comparator, Outcomes, and Study design). Inclusion criteria: Participants were adults (aged ≥18 years) exhibiting BP within the PHT range, defined as SBP between 120 and 139 mm Hg and/or DBP between 80 and 89 mm Hg (27). The intervention encompassed any mode of exercise training. Exercise interventions were classified into the following categories: aerobic exercise training [low-intensity training (LIT), moderate-intensity continuous training (MICT), and high-intensity interval training (HIIT)], isometric exercise training (IET), resistance training (RT), yoga, Tai Chi, or a combination of two or more of the above exercise forms. Supplementary Table S3 presents the detailed definitions of exercise types (3). Comparators included additional exercise modes or a non-exercise control group (usual care/health education) (4). The primary outcomes encompassed both SBP and DBP. Blood pressure can be measured using auscultatory, automatic, or 24-h ambulatory monitoring methods. FMD and PWV were utilized as outcomes for assessing endothelial function. Studies were required to incorporate measurements taken before and after the intervention (5). The study design was confined to RCTs, and the published language was restricted to English.

Exclusion criteria: (1) Non-randomized design; (2) Subjects with psychiatric disorders, cognitive impairment, pregnancy, cardiovascular diseases (e.g., heart failure, stroke, myocardial infarction), cancer, and pulmonary hypertension. People diagnosed with HTN, unless they included people with PHT whose results could be independently extracted. (3) Participants were receiving concurrent co-interventions attached to exercise (e.g., medication or dietary supplementation); (4) No detailed definition of the modalities of exercise; (5) Studies focusing exclusively on the acute assessment of BP; (6) For data derived from the same study population or duplicate published RCTs, we only included the one with the larger sample sizes and more comprehensive data; (7) The full text of the study or sufficient data [means and standard deviations (SD)] were unavailable through relevant databases and other sources, and the authors did not respond to our data requests; (8) Duplicate publications, reviews, systematic reviews, meta-analyses, conference abstracts, academic theses, letters, protocols, case reports, dissertations, and animal studies were excluded.

### 2.4 Eligibility assessment and data extraction

Search results were imported into NoteExpress software (version 3.7.0), where duplicates were removed. Two assessors independently examined the titles and abstracts to identify possibly relevant papers. If at least one investigator considered the paper eligible, a further full-text evaluation for final eligibility was obtained. Articles meeting the inclusion criteria after full-text screening were included in the NMA. Two investigators extracted the following data from each eligible article using a standardized form: article details (title, first author, publication year, country), characteristics of the study population (sample size, age, sex, disease status, treatment), outcome metrics (mean and SD at baseline and post-exercise or change values), specifics of exercise interventions (frequency, length, and duration), comparisons, and additional information. Origin 2024 was employed to extract the underlying numerical data from the graphical representation. Data consistency was verified through cross-checking of records following extraction. Discrepancies were addressed through discussion and, when necessary, the engagement of a third researcher.

### 2.5 Risk of bias assessment

Two researchers separately assessed the risk of bias for the included RCTs utilizing the updated Cochrane risk of bias assessment for randomized trials (RoB 2) (28–30). The criteria for evaluating bias across five areas: (1) randomized process, (2) allocation concealment, (3) missing outcome data, (4) assessment of outcomes, and (5) selection of the reported result. The risk of bias for each area and overall was categorized as low, some concerns, or high. Any disputes were settled through discussion or consultation with a third reviewer.

### 2.6 Data analysis

#### 2.6.1 Pairwise meta-analysis

Continuous data were synthesized as Mean Difference (MD) ± SD to evaluate and derive effect sizes for the interventions. Due to the clinical heterogeneity of the included trials, randomized pairwise meta-analyses were conducted utilizing the “meta” R package.

#### 2.6.2 Network meta-analysis

The Bayesian statistical model was developed with JAGS software version 4.3.1, incorporating the “gemtc” and “BUGSnet” packages (Rstudio, Boston, MA, USA). The results were analyzed using four Markov chains, each producing 50,000 iterations and burn-in at a step size of 20,000 pre-iterations. Network diagrams were generated by Stata SE 18.0 (StataCorp, College Station, Texas, USA). The convergence of the iterations was monitored using the Brooks-Gelman-Rubin approach, which analyzed within-chain and between-chain variation to calculate the Potential Scale Reduction Factor (PSRF). A PSRF close to 1 suggests that approximate convergence was achieved (23). We also compared and ranked the intervention types applying Surface Under the Cumulative Ranking curve (SUCRA) plots; an SUCRA value closer to 100% implies greater efficacy of the exercise (31). The I^2^ test was employed to evaluate statistical heterogeneity, with I^2^ values below 25% signifying low heterogeneity, values between 25% and 75% suggesting moderate heterogeneity, and values over 75% denoting high heterogeneity (32). We performed sensitivity analyses by eliminating articles lacking methodological rigor that exhibited a high risk of bias. In addition, the robustness of the results was evaluated by performing a meta-regression analysis with publication year, female ratio, mean age, treatment duration, and frequency as the covariates. Publication bias was assessed using comparison-adjusted funnel plots and Egger's test if ten or more trials were available for outcome comparison. Analyzes were deemed to exhibit publication bias when *p* < 0.05.

#### 2.6.3 Assessment of inconsistency

Fixed-effects and random-effects models were fitted using leverage-value plots along with the corresponding Deviation Information Criterion (DIC), effective number of parameters (pD), and deviance residuals (Dres). Lower values of DIC and Dres that approximate the total number of arms in the NMA indicate a better fit. A DIC difference of ≤3 suggests no significant difference between the models (33). Given the complex structure of evidence in NMA, the presence of evidence inconsistency needed to be assessed. DIC was also used to evaluate model consistency, with a DIC difference of ≤3 between the inconsistent model and the hypothetical consistent model indicating good consistency, and a larger difference suggesting inconsistency (34). Local inconsistency across direct, indirect, and network estimates was assessed using the node-splitting method, with inconsistency identified at *p* < 0.05 (35).

### 2.7 Certainty of evidence

The certainty of evidence for each outcome in the NMA was evaluated using the Confidence in Network Meta-Analysis (CINeMA) framework, which relies on the Grading of Recommendations Assessment, Development, and Evaluation (GRADE) methodology (36, 37). The assessment encompassed six domains: (1) within-study bias, (2) reporting bias, (3) indirectness, (4) imprecision, (5) heterogeneity, and (6) incoherence. Each domain was judged as having no, some, or major concerns. Similarly, being identical to the GRADE method, our confidence in the quality of evidence was rated as high, moderate, low, or very low for each relative intervention effect (38, 39).

### 2.8 Patient and public involvement

No patients were directly involved in this study's design, performance, measuring, and reporting. The findings from the NMA assessing physical activity will help inform the development of new exercise guidelines for PHT to prevent hypertension and associated illnesses.

## 3 Results

### 3.1 Literature screening process

**Figure 1:**
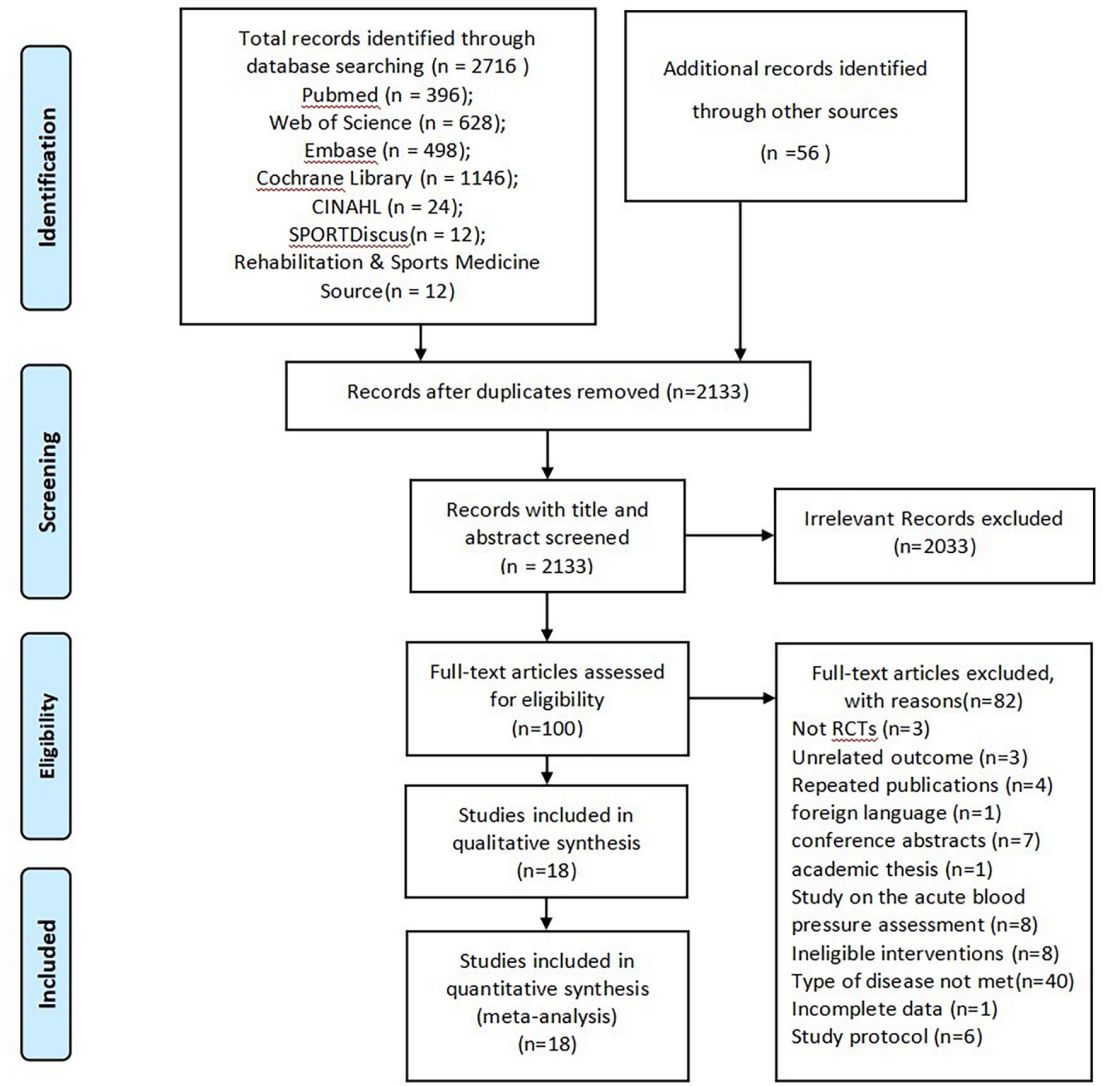
Flowchart of literature selection.

A total of 2,716 records were initially obtained through database searches, and an additional 56 entries were identified by reviewing reference lists. After eliminating duplicate records using NoteExpress, 2,133 articles were screened based on their titles and abstracts, and 2,033 records were discarded. The remaining 100 studies were downloaded for full-text evaluation. Among these, 3 articles were not RCTs, 3 studies reported unrelated outcomes, and 4 studies were duplicate publications. Additionally, 1 paper was in a foreign language, 7 studies were conference abstracts, 1 was an academic thesis, 8 trials focused on acute blood pressure assessment, 8 studies involved ineligible interventions, 40 studies included ineligible subjects, 1 study had incomplete data, and 6 studies were study protocols. The excluded full-text articles for reasons are presented in the Supplementary Table S4. Ultimately, 18 studies involving 2,538 subjects were included in this NMA. Figure 1 displays the PRISMA flow diagram for the literature selection process.

### 3.2 Study characteristics

Overall, 18 trials (21, 40–56) were eligible and included in our NMA. A total of 2,592 subjects were allocated to the control group (*n* = 830) and one or more of the following groups: 860 patients underwent aerobic exercise training (comprising 75 patients receiving LIT, 773 patients engaged in MICT, and 12 patients participating in HIIT), IET (*n* = 226), RT (*n* = 130), Yoga (*n* = 250), Tai Chi (*n* = 173), and a combination of aerobic exercise with RT (*n* = 123). The patients' ages varied from 19.0 to 67.3 years. Regarding publishing locations, 5 studies were conducted in the USA (41, 42, 45, 49, 56); 4 studies in India (44, 48, 53, 55); 2 studies in Thailand (52, 54); and 1 research each in China (46); Nigeria (51); UK (50); Kenya (47); Malaysia (43); Iran (40); and Chile (21). The exercise duration spanned from 24 days to 1 year, the frequency ranged from 3 to 7 times per week, and the duration of a single session ranged from 15 to 60 min. AT mainly consists of treadmill, cycling, walking, and swimming. Tai Chi is represented by 24-form Yang-style Tai Chi. IET consists of isometric handgrip and wall squat exercise training, with intensity measured by 30% maximum voluntary contraction or 95% heart rate peak. RT intensity ranged from 20 to 80% one-repetition maximum.The outcome of SBP was reported in 17 trials (40–56), DBP in 17, FMD in 5 (21, 40–42, 49), and PWV in 5 (21, 41, 49, 54, 56). More details of the publications included are summarized Table 1.

**Table 1:** Characteristics of included studies.

Author year	Country	Type	Population	Sample size (female)	Average age	Exercise intervention	Frequency (days/week)	Exercise intensity	Exercise duration	Outcome measure
Alvarez et al., 2024	Chile	RCT	Adults	CON: 10 HIIT + RT: 8	CON: 34.2 ± 11.2 HIIT + RT: 50.3 ± 14.8	15 min for HIIT (cycling) and 10 min for RT	3	HIIT: 80%–100% HRpeak RT: 20%–50% 1RM	6 weeks	FMD, PWV
Azadpour et al., 2017	Iran	RCT	Obese postmenopausal women	CON: 12 (12) MICT:12 (12)	CON: 56.58 ± 4.17 MICT: 57.58 ± 4.29	25–40 min treadmill walking/running	3	MICT: 50%–70% HRR	10 weeks	SBP, DBP, FMD
Banks et al., 2024	USA	RCT	Middle-aged and older adults (age 45–64 years)	CON: 13 (8) RT: 13 (8)	CON: 52 ± 6 RT: 55 ± 6	40 min bench press, hack squat, seated row, etc.	3	NR	9 weeks	SBP, DBP, FMD, PWV
Beck et al., 2013	USA	RCT	Young adults (age 18–35 years)	CON: 15 (5) AT: 13 (4) RT: 15 (4)	CON: 21.6 ± 2.9 AT: 20.1 ± 1.1 RT: 21.1 ± 2.5	AT: 60 min treadmill walking/running RT: 60 min 8–12 repetitions on variable resistance machines	3	AT: 65%–85% HRpeak RT: 50% 1RM	8 weeks	SBP, DBP, FMD
John et al., 2022	Malaysia	RCT	Young adults (age 18–25 years)	CON: 12 (6) HIIT: 10 (4) MICT: 13 (4)	CON: 21 ± 1.0 HIIT: 21 ± 0.8 MICT: 19 ± 1.3	HIIT: 1:4 min work to rest ratio on treadmill MICT: treadmill continuously for 20 min	3	HIIT: 80%–85% HRR MICT: 40%–60% HRR	4 weeks	SBP, DBP
Krishna et al., 2023	India	RCT	Adults (age 18–35 years)	CON:39 (18) Yoga: 32 (14)	CON: 18.69 ± 0.65 Yoga: 18.69 ± 0.82	Yoga therapy for 60 min	3	NR	12 weeks	SBP, DBP
Lee et al., 2024	USA	RCT	Overweight or obesity adults (age 35–70 years)	CON: 102 (55) RT: 102 (53) AT: 101 (53) AT + RT: 101 (55)	CON: 50 ± 10 RT: 50 ± 10 AT: 51 ± 10 AT + RT: 50 ± 10	RT: 12 weight-lifting machines, complete each set to fatigue, 60 min AT: treadmills, stationary bicycles, and elliptical machines, 60 min RT + AT: 30 min RT combine with 30 min AT	3	AT at 50%–80% HRR and RT at 50%–80% 1RM	1 year	SBP, DBP
Li et al., 2024	China	RCT	Adults (age 18–65 years)	Tai Chi: 173 (87) AT: 169 (89)	Tai Chi: 48.4 ± 12.4 AT: 50.1 ± 11.4	Tai Chi: 24-form Yang-style Tai Chi for 60 min AT: climbing stairs, jogging, brisk walking, and cycling for 60 min	4	AT: maximum heart rate was estimated as 208−(0.7 × age in years)	12 months	SBP, DBP
Magutah et al., 2022	Kenya	RCT	Sedentary adults (age 18–79 years)	CON:182 (101) AT1: 231 (130) AT2: 252 (140)	CON: 34.87 ± 11.59 AT1: 35.35 ± 12.78 AT2: 34.28 ± 11.55	AT1: 3 bouts of 7.5 min jogging and related activities AT2: 30–60 min jogging and related activities	AT1: 7 AT2: 3	AT: 50%–70% HRpeak	12 weeks	SBP, DBP
Narnolia et al., 2024	India	RCT	Adults (age 30–60 years)	Yoga: 50 (11) Walking: 50 (11)	Yoga: 43.02 ± 7.24 Walking: 45.36 ± 6.24	Sudarshan Kriya Yoga 60 min	7	NR	12 weeks	SBP, DBP
Nualnim et al., 2022	USA	RCT	Adults (age 50–80 years)	CON: 19 (15) Swimming: 24 (17)	CON: 61 ± 2 Swimming: 58 ± 2	First few weeks: swim 15–20 min/day, 3–4 days/week; last few weeks: 40–45 min/day, 3–4 days/week	3–4	70%–75% of HRpeak	12 weeks	SBP, DBP
O'Driscoll et al., 2022	UK	RCT	Male adults	CON: 12 (0) IET: 12 (0)	45 (41.3, 49)	Wall squat IET sessions, 4 × 2 min bouts of isometric wall squat, separated with 2-min rest intervals	3	95% HRpeak	1 year	SBP, DBP
Ogbutor et al., 2019	Nigeria	RCT	Adults	CON: 200 (88) IET: 200 (91)	CON: 41.27 ± 6.31 IET: 40.78 ± 6.04	Isometric handgrip exercise training at 30% maximum voluntary contraction for 2 min	7	30% maximum voluntary contraction	24days	SBP, DBP
Prasertsri et al., 2019	Thailand	RCT	Sedentary adults (age 60–80 years)	CON: 25 (21) LIT: 25 (18)	CON: 67.32 ± 6.89 LIT: 67.24 ± 5.29	Both arms swing forward to around 30 and backward to around 60 slowly	3	Low-intensity exercise based on ∼23% of VO2peak or ∼45% of HRpeak	12 weeks	SBP, DBP
Singh et al., 2022	India	RCT	Adults	CON: 121 (53) Yoga: 117 (67)	CON: 49.3 ± 10.7 Yoga: 50.3 ± 9.9	Yoga therapy for 60 min	4		6 months	SBP, DBP
Songcharern et al., 2022	Thailand	RCT	Young sedentary men with hypertensive parents (age 18–22 years)	CON: 15 (0) AT + RT: 14 (0)	CON: 19.5 ± 0.5 AT + RT: 19.6 ± 1.7	5 min of warm-up, 20 min of RT (seated rowing machine, bicep curls, and squats, etc.), 30 min of treadmill walking, and 5 min of static stretching	3	AT: 50%–60% HRR in weeks 1–4, to 60%–70% HRR in weeks 5–8 RT: 50%–70% of 1RM during weeks 1–4, 60%–80% of 1RM during weeks 5–8	8 weeks	SBP, DBP, PWV
Thiyagarajan et al., 2015	India	RCT	Adults (age 20–60 years)	CON: 15 (0) Yoga: 14 (0)	CON: 42.47 ± 9 Yoga: 44.08 ± 9.42	Yoga therapy for 45 min	3	NR	12 weeks	SBP, DBP
Wong et al., 2020	USA	RCT	Young obese female (age 19–27 years)	CON: 14 (14) Pilates training: 14 (14)	CON: 23 ± 1 Pilates training: 22 ± 1	Warm up (10 min), general conditioning consisting of Pilates exercises (40 min), and stretching and cooling down (10 min)	3	NR	12 weeks	SBP, DBP, PWV

AT, aerobic training; CON, control group; DBP, diastolic blood pressure; FMD, flow-mediated dilation; HIIT, high-intensity interval training; HRpeak, peak heart rate; HRR, heart rate reserve; IET, isometric exercise training; LIT, low intensity training; MICT, moderate-intensity continuous training; NR, not reported; PWV, pulse wave velocity; RCT, random controlled trial; RT, resistance training; SBP, systolic blood pressure; 1RM, one-repetition maximum.

### 3.3 Risk of bias

**Figure 2:**
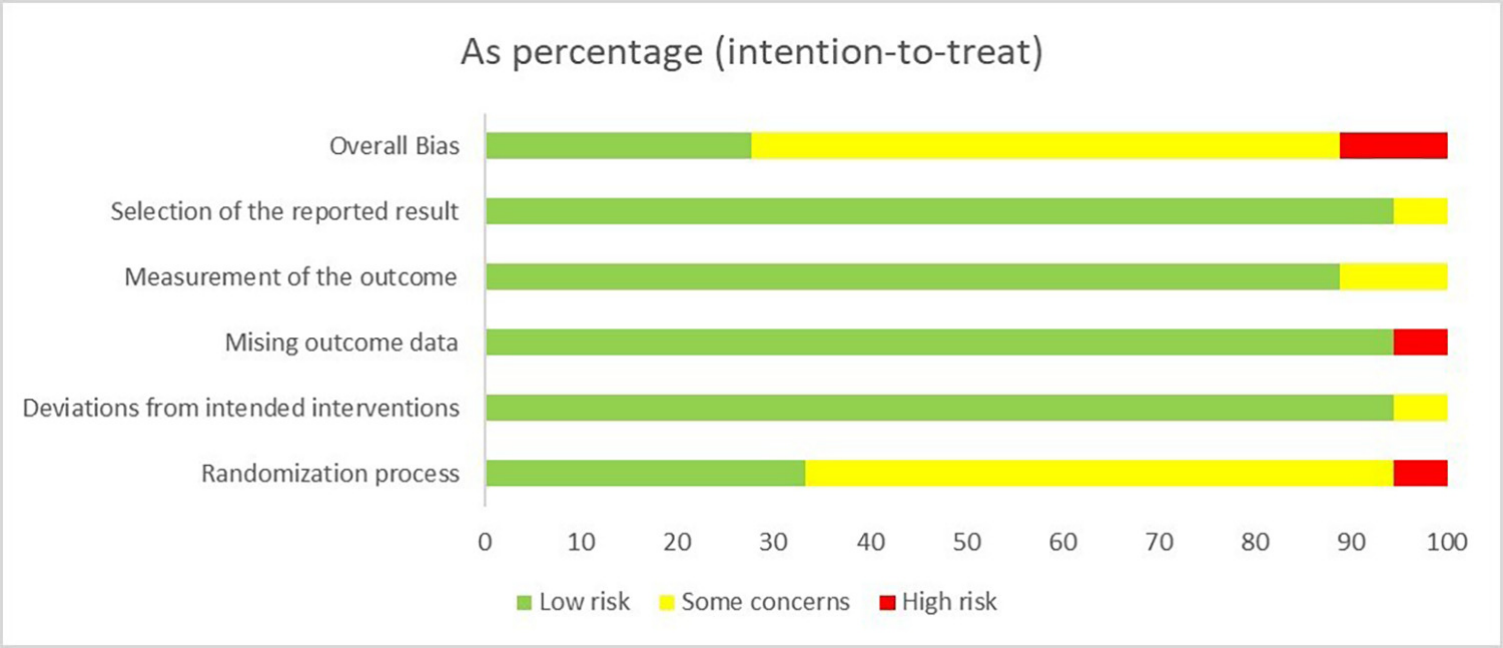
Summary of risk-of-bias assessment.

Figure 2 summarizes the results of the risk of bias according to ROB2. Of 18 included RCTs, 5 trials (27.8%) were rated low risk (45–47, 51, 56), 11 trials (61.1%) were judged to have some concerns (40–44, 48–50, 52–54), and 2 (11.1%) study at high risk (21, 55). The high risk of bias is mainly attributed to the unbalanced randomization process and bias from missing outcome data. Among all the studies, 16 trials (88.9%) reported on the randomization process (21, 40–43, 45–48, 50–56) while only 6 trials (33.3%) adequately concealed the intervention allocation (45–47, 51, 55, 56). Owing to the characteristics of exercise therapies, blinding patients and researchers prove challenging, with only 4 (22.2%) reporting outcome assessor blinding (40, 45, 46, 55). Supplementary Figure S1 presents detailed information on the ROB2 evaluation for all included studies. Future high-quality RCTs are necessary for this area and should be improved, especially at the methodological level of allocation concealment, blinding, and missing data handling, to provide a more robust evidence-based basis.

### 3.4 Network geometry

**Figure 3:**
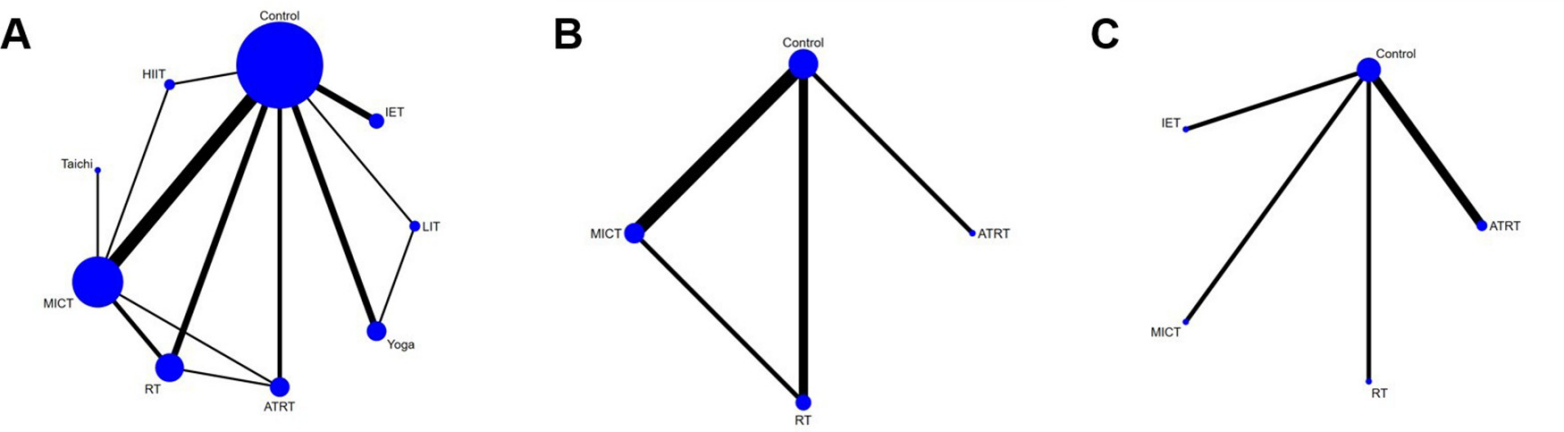
Network plots for **(A)** SBP and DBP, **(B)** FMD, and **(C)** PWV.

The network geometry graph displays the treatment comparisons of different exercise modalities for SBP, DBP (Figure 3A), FMD (Figure 3B), and PWV (Figure 3C). The SBP and DBP NMA (17 studies, *n* = 2574 patients) consisted of 9 conditions, 13 direct pairwise comparisons, and 34 indirect pairwise comparisons. The FMD NMA (5 studies, *n* = 154 patients) consisted of 4 conditions, 4 direct pairwise comparisons, and 5 indirect pairwise comparisons. The PWV NMA (5 studies, *n* = 144 patients) consisted of 5 conditions, 4 direct pairwise comparisons, and 6 indirect pairwise comparisons.

### 3.5 Systolic blood pressure

The comparative impact of any exercise prescription vs. control on SBP is illustrated in Supplementary Figure S2.1. The posterior MD for all exercise training varied from −0.38 to −8.67 mm Hg, with the most substantial network estimate attributed to Tai Chi [−8.67 mm Hg, 95% credible interval (CI) (−17.29 to −0.05, p_random_ = 0.04)]. Compared with control, the effect of HIIT [−6.47 mm Hg, 95% CI (−13.92–0.99), p_random_ = 0.09], ATRT [−5.28 mm Hg, 95% CI (−10.87–0.31), p_random_ = 0.06], and LIT [−0.38 mm Hg, 95% CI (−6.86–6.10), p_random_ = 0.91] were the comparisons that were not statistically significant.

### 3.6 Diastolic blood pressure

The comparative impact of any exercise prescription vs. control on DBP is shown in Supplementary Figure S2.2. The posterior MD for all exercise training varied from −0.54 to −4.61 mm Hg, with the most substantial network estimate from IET at −4.61 mm Hg (95% CI: −8.11 to −1.11, p_random_ = 0.01). In comparison to the control group, the effects of Tai Chi [−4.49 mm Hg, 95% CI (−10.59–1.62), p_random_ = 0.15], HIIT [−3.76 mm Hg, 95% CI (−9.95–2.43), p_random_ = 0.23], and LIT [−0.54 mm Hg, 95% CI (−5.13–4.06), prandom = 0.82] were the comparisons that were not statistically significant.

### 3.7 Flow-mediated dilation

The comparative impact of any exercise prescription vs. control on FMD is shown in Supplementary Figure S2.3. The posterior MD for all exercise training varied from 2.66% to 5.82%, with each exercise modality revealing a significant difference in FMD improvement. The most effective network estimates were obtained from MICT [5.82%, 95% CI (5.41–6.22), p_random_ < 0.01], followed by ATRT [5.60%, 95% CI (0.53–10.67), p_random_ = 0.03], and RT [2.66%, 95% CI (1.11–.21), p_random_ < 0.001].

### 3.8 Pulse wave velocity

The comparative impact of any exercise prescription vs. control on PWV is illustrated in Supplementary Figure S2.4. The posterior MD for all exercise training varied from −0.10 to −0.82 m/s, with the most substantial network estimates obtained from IET [−0.82 m/s, 95% CI (−1.58 to −0.06), p_random_ = 0.03]. Compared to the control, the effects of ATRT [−0.59 m/s, 95% CI (−1.33–0.14), p_random_ = 0.11], MICT [−0.25 m/s, 95% CI (−0.92–0.42), p_random_ = 0.46], and RT [−0.10 m/s, 95% CI (−0.88–0.68), p_random_ = 0.80] were statistically non-significant.

### 3.9 Ranking superiority and NMA estimates

**Figure 4:**
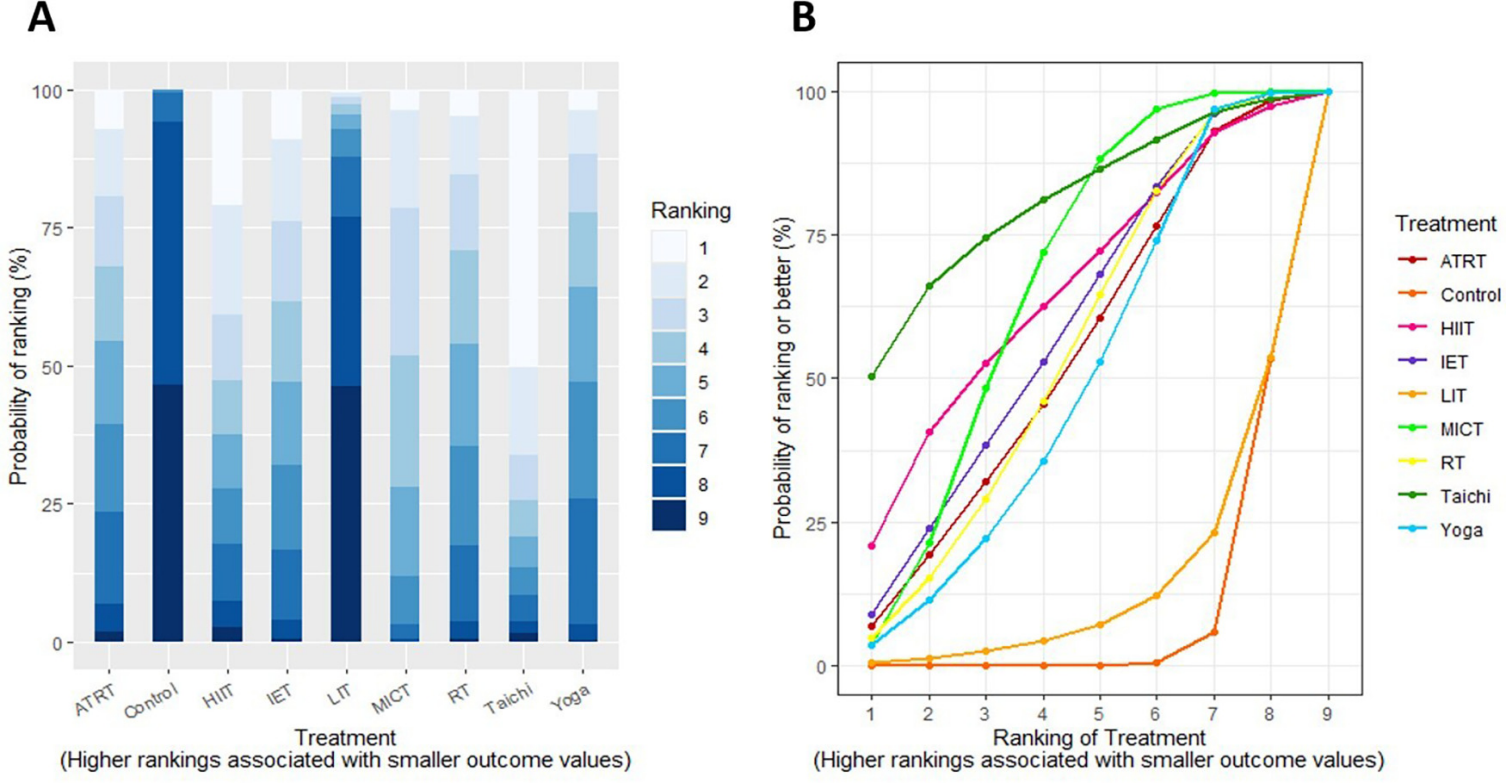
**(A)** treatment rank probabilities plot for SBP and **(B)** SUCRA plot for SBP.

**Figure 5:**
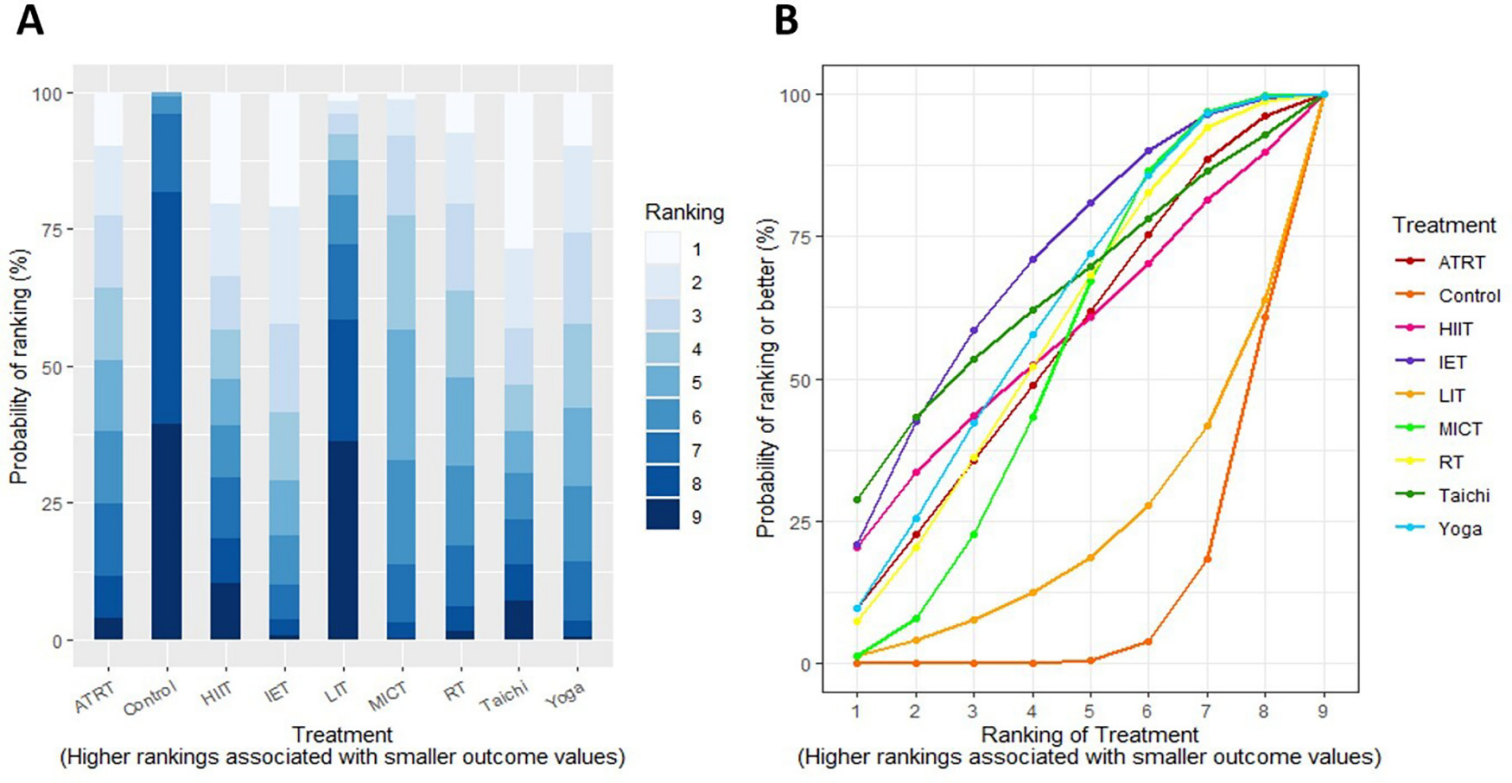
**(A)** treatment rank probabilities plot for DBP and **(B)** SUCRA plot for DBP. Two charts compare treatments based on ranking probability.

We conducted a treatment rank probability analysis to ascertain the ranking of all interventions. Additionally, we produced SUCRA plots to visualize the probability percentages of ranking. Figure 4 displays the treatment rank plot and SUCRA plot for SBP, with the top three ranked exercises being Tai Chi (79%), followed by MICT (66%), and HIIT (64%). League plots (Supplementary Figure S3) were created to provide a detailed summary of the posterior MD and 95% CIs for SBP from the NMA, illustrating the significance of each exercise prescription in comparison to the control group and other interventions. Within these plots, green units indicate superior treatment efficiency compared to the comparator, whereas red units suggest inferior performance. Statistical differences at the 95% CI between interventions and their comparators are marked with (**). The primary findings for SBP showed that Tai Chi was significantly effective in reducing SBP compared to MICT [−2.40 mm Hg, 95% CI (−4.37 to −0.42)], while IET also demonstrated a significant superiority over MICT [−2.16 mm Hg, 95% CI (−2.65 to −1.69)]. Figure 5 displays the treatment rank plot and SUCRA plot for DBP, with the leading three ranked exercises being IET (71%), followed by Tai Chi (66%), and Yoga (62%). The principal findings for DBP indicated that IET was significantly more effective than Yoga [−1.44 mm Hg, 95% CI (−2.03 to −0.87)]. Comparative details regarding other exercise modalities are available in Supplementary Figure S4. For FMD, the leading three exercises ranked were MICT (84%), ATRT (77%), and RT (38%) (Supplementary Figure S5). Comparatively, MICT was significantly superior at improving FMD than RT [−3.16 mm Hg, 95% CI (−1.57 to −4.74)]. No other notable differences in FMD were observed among the various exercise therapies. The top three exercises for PWV were IET (85%), ATRT (71%), and MICT (44%) (Supplementary Figure S6). IET demonstrated significantly better efficacy in decreasing PWV compared to MICT [−0.57 mm Hg, 95% CI (−0.98 to −0.16)] and RT [−0.72 mm Hg, 95% CI (−1.29 to −0.14)]. In contrast, no significant differences in PWV were observed among the other exercise therapies.

### 3.10 Model fits and network inconsistency

In terms of modeling fit, Supplementary Figure S7 provides the plots of leverage values, which show the corresponding Dres and DIC. The random effects models for SBP and DBP exhibited lower DIC, and the leverage values indicated fewer outliers, thus the random effects models were selected. No significant differences were observed across the model fit parameters for FMD and PWV. Regarding the consistency assessment, the DIC comparisons showed good agreement for SBP (DIC, 73.97 vs. 74.51), DBP (DIC, 74.12 vs. 74.87), FMD (DIC, 19.54 vs. 19.59), and PWV (DIC, 19.58 vs. 19.57). Nodal split analyses of the primary results indicated no significant differences among direct, indirect, and NMA evidence (Bayesian *p* > 0.05), implying no local inconsistency exists (Supplementary Figure S8). The PSRF values in all comparisons approached 1.00, suggesting that approximate convergence had been achieved.

### 3.11 Sensitivity analyses and meta-regressions

Sensitivity analyses were performed for the primary outcome by excluding research with a high risk of bias. The results were generally consistent following the original NMA, except that the effect of the Yoga intervention on SBP was changed to −5.96 mm Hg with 95% CI (−11.1 to −0.81) (Supplementary Figure S9). Furthermore, meta-regression analyses revealed that covariates (publication year, female ratio, mean age, treatment duration, and frequency) did not influence the results of this NMA (Supplementary Table S5).

### 3.12 Publication bias

Studies containing SBP and DBP outcome indicators exceeding 10 were assessed for publication bias using funnel plots (Supplementary Figure S10). The comparison-adjusted funnel plots show that all studies are predominantly distributed on either side of the zero line, indicating no significant asymmetry. Egger's test results for SBP and DBP exhibited no significant publication bias (p_SBP_ = 0.59, p_DBP_ = 0.79). Nevertheless, several studies remained outside the boundaries of the 95% CI, suggesting moderate to substantial heterogeneity among these studies.

### 3.13 Certainty of evidence

Supplementary Table S6 displays the detailed results derived from the CINeMA approach for NMA. The quality of evidence for SBP and DBP varied from moderate to very low; FMD outcomes were rated as very low, and PWV outcomes ranged from moderate to very low. For primary outcomes, the effect of Tai Chi on SBP demonstrated low certainty mainly due to imprecision, which was related to the small sample size in the included papers. The findings of IET on DBP were downgraded to moderate to very low quality primarily by heterogeneity (I^2^ ≥ 50%) and imprecision. The confidence of the MICT evidence on FMD was rated as very low, and all of the evidence was downgraded by within-study bias and imprecision. Evidence involving the effect of IET on PWV was rated as moderate quality.

## 4 Discussion

To our knowledge, this systematic review and NMA provide the most comprehensive synthesis of data on different exercise interventions in prehypertensive populations. This NMA evaluated all relevant RCTs identified through systematic searches, comprising 17 trials and 2,574 participants, to assess the efficacy and superiority of diverse exercise programs in lowering blood pressure, enhancing endothelial function, and reducing vascular stiffness. Pairwise analysis indicated that most exercise modalities significantly reduced blood pressure and FMD compared to controls, while statistical significance for PWV was observed only with IET. NMA results suggest that Tai Chi is the most efficacious exercise for reducing SBP, followed by MICT and HIIT in second and third place, respectively. For DBP, IET exhibited the most significant MD, succeeded by Tai Chi and Yoga. Regarding vascular function, IET was considered the most effective exercise for decreasing PWV, whereas MICT showed the best efficacy in improving FMD.

After excluding studies at high risk of bias, the sensitivity analysis results were generally consistent with the original NMA, demonstrating the robustness of our findings. Previous studies have found that exercise durations exceeding 12 weeks and frequency exceeding 5 times per week are associated with better blood pressure reductions (57). Younger age may lead to greater improvements in vascular function (58). A prospective study involving 412,413 U.S. adults has also revealed that women could derive greater all-cause and cardiovascular mortality risk reduction benefits from equivalent doses of physical activity compared with men (59). However, in the meta-regression of our study, no significant effect of year of publication, female ratio, mean age, intervention duration, and frequency on the outcome of the NMA has been identified, which may be related to the small number of RCTs included in this NMA, and these findings should be treated with caution. The majority of studies showed some concern regarding “within-study bias”, essentially due to flaws in the randomized concealment process and blinding by exercise interventions. “Imprecision” and “heterogeneity” were also the principal factors for downgrading in most comparisons, which partially affects the credibility of the results.

Currently, most meta-analyses examining the effects of exercise types on BP concentrate on general populations or mixed samples of hypertensive and prehypertensive individuals. A previous large-scale NMA reported that IET was the most effective approach for improving BP (60), whose results for SBP were inconsistent with our findings. This disparity may be ascribed to their inclusion of hypertensive and normotensive individuals and that our NMA incorporated more recent research. Our study focuses on the PHT population and is the first to include Tai Chi as a novel exercise modality in NMA alongside established methods like MICT, RT, and IET. Tai Chi, a traditional Chinese mind-body practice, emphasizes the combination of movement, mindfulness, and respiration (61). This holistic exercise form could further enhance its efficacy in reducing SBP by alleviating stress and improving autonomic nervous system function (62). Previous studies have confirmed that healthy lifestyle modifications (body mass index, diet, smoking, alcohol consumption, sodium excretion, and sedentary behavior) can reduce SBP by 3.5 mm Hg (63). For every 1 mm Hg decrease in SBP, the risk of cardiovascular disease declined by approximately 2%–3% (64). The mean difference in Tai Chi reduction in our NMA was up to −8.67 mm Hg, which may have clinical significance for preventing cardiovascular disease or even reducing the risk of cardiovascular events. Li et al. (46) confirmed that Tai Chi exhibits significant advantages over conventional MICT in decreasing BP load, implying a stronger capacity to reduce hypertension risk (65). Although imprecision has resulted in the effect of tai chi on SBP exhibiting low confidence of evidence, this may be attributed to the new emergence of the PHT definition in recent years and the insufficient sample size of studies conducted for this area. As far as the existing literature is concerned, the effect of tai chi on BP is unquestionable (66). A recent meta-analysis also confirmed that Tai Chi can more significantly reduce SBP in hypertensive patients compared to regular walking or aerobic exercise (67). Our investigation into the comparative effects of different exercise modalities revealed that both Tai Chi and IET surpassed MICT in lowering SBP, further supporting their potential advantages in BP reduction.

In recent years, several studies have shown that IET has a more effective antihypertensive effect than aerobic exercise (68, 69). This applies not only to hypertensive individuals but also demonstrates superior advantages in PHT individuals with lower baseline BP levels. O'Driscoll et al. (50) observed that PHT participants experienced reductions of 10.5 mm Hg in SBP and 8 mm Hg in DBP following a 1-year IET intervention. IET, characterized by sustained muscle contractions without changes in muscle length, involves highly complex mechanisms to reduce BP. Existing studies indicate that the primary mechanisms for IET's hypotensive effects are decreasing total peripheral resistance and auguring vascular compliance (70). Our NMA partially supports that perspective. Compared to the control group, IET is the only exercise modality with a significant effect on PWV, achieving the highest rank in the NMA and corroborated by moderate-quality evidence. However, more high-quality evidence is still required to support it in the future, owing to the limited number of studies.

Our findings indicate that all exercise types can effectively enhance vascular endothelial function in PHT individuals, with MICT producing the most notable benefits. A 1% rise in FMD corresponded to a 13% reduction in cardiovascular risk (71). In our NMA, MICT was associated with a mean 5.82% increase in FMD, indicating that MICT can significantly improve endothelial function in patients with PHT, potentially reducing cardiovascular events. Studies suggest that aerobic exercise improves endothelial function by elevating arterial wall shear stress via recurrent perfusion, reducing reactive oxygen species, and upregulating endothelial nitric oxide synthase (eNOS) and nitric oxide (NO) (72, 73). We obtained different ranking results in evaluating FMD outcome relative to PWV, presumably owing to inadequate trial and sample size data. Although MICT was identified as the most efficacious modality for improving FMD, the absence of IET, Tai Chi, and HIIT from this NMA prevents a comprehensive comparison of exercise options. Consequently, additional clinical studies are required and recommended to examine the antihypertensive effects of various exercise modalities mediated through vascular function, thus providing more robust scientific evidence and practical guidance for the prevention and management of PHT. This would help mitigate the prevalence of HTN and associated cardiovascular complications, improving long-term health outcomes for PHT patients.

## 4 Limitations

It should be acknowledged that several limitations may have impacted the results of this study. First, the risk of bias assessment revealed several methodological shortcomings in the included trials, including deficiencies in random allocation concealment, absence of blinding for exercise interventions, and missing data attributed to low adherence. Secondly, the evidence is inherently unstable due to the limited number of RCTs included in this NMA. Future research should focus on conducting high-quality, large-scale RCTs to strengthen the evidence base. Thirdly, because of data and sample size restrictions, we were unable to identify the optimal dosage parameters. Therefore, additional definitive studies are required to investigate various intensities, durations, and frequencies of effective exercise types to clarify guidelines. Fourth, the majority of evidence was evaluated as low or very low quality, with downgraded confidence levels owing to risks of bias and heterogeneity. Despite performing sensitivity analyses and meta-regressions, we could not identify the principal causes of heterogeneity. Therefore, these findings need to be interpreted with prudence.

## 5 Conclusion

This systematic review and NMA demonstrate the significant effect of exercise on BP and vascular function in PHT individuals. Tai Chi exhibits the greatest effectiveness in reducing SBP, whereas IET produces the most notable improvements in DBP and PWV. MICT stands out in enhancing endothelial function. These findings provide a scientific basis for developing personalized exercise prescriptions. Particularly, Tai Chi and IET, owing to their proven efficacy and practicality, merit broader adoption in clinical and public health contexts. Nevertheless, further high-quality studies encompassing diverse exercise modalities are necessary to refine optimal exercise strategies and dosing parameters, thereby enhancing guidance for PHT prevention and management.

## Data Availability

The original contributions presented in the study are included in the article/Supplementary Material, further inquiries can be directed to the corresponding author.
