# Supplementary material for: Effects of different exercise modalities on blood pressure and endothelial function in prehypertension individuals: a systematic review and network meta-analysis
Source: Front Cardiovasc Med. 2025 Jun 25;12:1550435. doi: 10.3389/fcvm.2025.1550435 (PMC12239222; doi:10.3389/fcvm.2025.1550435)
Supplement: Supplementary file 1 [file Datasheet1.docx]

Supplementary Material

**1 Supplementary Figures**

**Supplementary Figure1. Detailed information of the risk of bias assessment for each eligible study**

**Supplementary Figure2 Forest plot of each modality of exercise compared to Control**

**Supplementary Figure3. League heat plot for SBP**

**Supplementary Figure4. League heat plot for DBP**

**Supplementary Figure5 Ranking superiority and NMA estimates for FMD**

**Supplementary Figure6 Ranking superiority and NMA estimates for PWV**

**Supplementary Figure7 Fixed and random effects modeling**

**Supplementary Figure8. Node-splitting analysis**

**Supplementary Figure9. Sensitivity analysis of the primary outcomes**

**Supplementary Figure10. Plot of funnel**

**2 Supplementary Tables**

**Supplementary Table 1. PRISMA checklist**

**Supplementary Table 2. Specific search strategies for each database**

**Supplementary Table 3. Categories of exercise modalities**

**Supplementary Table 4. Excluded full-text with reasons**

**Supplementary Table5. Meta-regression analysis results**

**Supplementary Table6. CINeMA confidence rating**

**Supplementary Table 1. PRISMA checklist**

| **Section and Topic** | **Item #** | **Checklist item** |
| --- | --- | --- |
| **TITLE** | | |
| Title | 1 | Identify the report as a systematic review. |
| **ABSTRACT** | | |
| Abstract | 2 | See the PRISMA 2020 for Abstracts checklist. |
| **INTRODUCTION** | | |
| Rationale | 3 | Describe the rationale for the review in the context of existing knowledge. |
| Objectives | 4 | Provide an explicit statement of the objective(s) or question(s) the review addresses. |
| **METHODS** | | |
| Eligibility criteria | 5 | Specify the inclusion and exclusion criteria for the review and how studies were grouped for the syntheses. |
| Information sources | 6 | Specify all databases, registers, websites, organisations, reference lists and other sources searched or consulted to identify studies. Specify the date when each source was last searched or consulted. |
| Search strategy | 7 | Present the full search strategies for all databases, registers and websites, including any filters and limits used. |
| Selection process | 8 | Specify the methods used to decide whether a study met the inclusion criteria of the review, including how many reviewers screened each record and each report retrieved, whether they worked independently, and if applicable, details of automation tools used in the process. |
| Data collection process | 9 | Specify the methods used to collect data from reports, including how many reviewers collected data from each report, whether they worked independently, any processes for obtaining or confirming data from study investigators, and if applicable, details of automation tools used in the process. |
| Data items | 10a | List and define all outcomes for which data were sought. Specify whether all results that were compatible with each outcome domain in each study were sought (e.g. for all measures, time points, analyses), and if not, the methods used to decide which results to collect. |
|  | 10b | List and define all other variables for which data were sought (e.g. participant and intervention characteristics, funding sources). Describe any assumptions made about any missing or unclear information. |
| Study risk of bias assessment | 11 | Specify the methods used to assess risk of bias in the included studies, including details of the tool(s) used, how many reviewers assessed each study and whether they worked independently, and if applicable, details of automation tools used in the process. |
| Effect measures | 12 | Specify for each outcome the effect measure(s) (e.g. risk ratio, mean difference) used in the synthesis or presentation of results. |
| Synthesis methods | 13a | Describe the processes used to decide which studies were eligible for each synthesis (e.g. tabulating the study intervention characteristics and comparing against the planned groups for each synthesis ). |
|  | 13b | Describe any methods required to prepare the data for presentation or synthesis, such as handling of missing summary statistics, or data conversions. |
|  | 13c | Describe any methods used to tabulate or visually display results of individual studies and syntheses. |
|  | 13d | Describe any methods used to synthesize results and provide a rationale for the choice(s). If meta-analysis was performed, describe the model(s), method(s) to identify the presence and extent of statistical heterogeneity, and software package(s) used. |
|  | 13e | Describe any methods used to explore possible causes of heterogeneity among study results (e.g. subgroup analysis, meta-regression). |
|  | 13f | Describe any sensitivity analyses conducted to assess robustness of the synthesized results. |
| Reporting bias assessment | 14 | Describe any methods used to assess risk of bias due to missing results in a synthesis (arising from reporting biases). |
| Certainty assessment | 15 | Describe any methods used to assess certainty (or confidence) in the body of evidence for an outcome. |
| **RESULTS** | | |
| Study selection | 16a | Describe the results of the search and selection process, from the number of records identified in the search to the number of studies included in the review, ideally using a flow diagram. |
|  | 16b | Cite studies that might appear to meet the inclusion criteria, but which were excluded, and explain why they were excluded. |
| Study characteristics | 17 | Cite each included study and present its characteristics. |
| Risk of bias in studies | 18 | Present assessments of risk of bias for each included study. |
| Results of individual studies | 19 | For all outcomes, present, for each study: (a) summary statistics for each group (where appropriate) and (b) an effect estimate and its precision (e.g. confidence/credible interval), ideally using structured tables or plots. |
| Results of syntheses | 20a | For each synthesis, briefly summarise the characteristics and risk of bias among contributing studies. |
|  | 20b | Present results of all statistical syntheses conducted. If meta-analysis was done, present for each the summary estimate and its precision (e.g. confidence/credible interval) and measures of statistical heterogeneity. If comparing groups, describe the direction of the effect. |
|  | 20c | Present results of all investigations of possible causes of heterogeneity among study results. |
|  | 20d | Present results of all sensitivity analyses conducted to assess the robustness of the synthesized results. |
| Reporting biases | 21 | Present assessments of risk of bias due to missing results (arising from reporting biases) for each synthesis assessed. |
| Certainty of evidence | 22 | Present assessments of certainty (or confidence) in the body of evidence for each outcome assessed. |
| **DISCUSSION** | | |
| Discussion | 23a | Provide a general interpretation of the results in the context of other evidence. |
|  | 23b | Discuss any limitations of the evidence included in the review. |
|  | 23c | Discuss any limitations of the review processes used. |
|  | 23d | Discuss implications of the results for practice, policy, and future research. |
| **OTHER INFORMATION** | | |
| Registration and protocol | 24a | Provide registration information for the review, including register name and registration number, or state that the review was not registered. |
|  | 24b | Indicate where the review protocol can be accessed, or state that a protocol was not prepared. |
|  | 24c | Describe and explain any amendments to information provided at registration or in the protocol. |
| Support | 25 | Describe sources of financial or non-financial support for the review, and the role of the funders or sponsors in the review. |
| Competing interests | 26 | Declare any competing interests of review authors. |
| Availability of data, code and other materials | 27 | Report which of the following are publicly available and where they can be found: template data collection forms; data extracted from included studies; data used for all analyses; analytic code; any other materials used in the review. |

*From:*  Page MJ, McKenzie JE, Bossuyt PM, Boutron I, Hoffmann TC, Mulrow CD, et al. The PRISMA 2020 statement: an updated guideline for reporting systematic reviews. BMJ 2021;372:n71. doi: 10.1136/bmj.n71

For more information, visit: <http://www.prisma-statement.org/>

**Supplementary Table 2. Specific search strategies for each database**

| Database | Search Terms | Result |
| --- | --- | --- |
| **Pubmed (Date: September 12, 2024)** | | |
| No. |  | |
| #1 | "prehypertension"[MeSH Terms] | 1,209 |
| #2 | "prehypertension*"[Title/Abstract] OR "pre hypertension*"[Title/Abstract] OR "pre hypertension*"[Title/Abstract] OR "Pre-HTN"[Title/Abstract] OR "PreHTN"[Title/Abstract] OR "high normal blood pressure"[Title/Abstract] OR "elevated blood pressure"[Title/Abstract] OR "raised blood pressure"[Title/Abstract] OR "borderline hypertension*"[Title/Abstract] OR "early hypertension*"[Title/Abstract] | 13,183 |
| #3 | #1 OR #2 | 13,388 |
| #4 | "exercise"[MeSH Terms] OR "resistance training"[MeSH Terms] OR "endurance training"[MeSH Terms] OR "high intensity interval training"[MeSH Terms] OR "walking"[MeSH Terms] OR "jogging"[MeSH Terms] | 286,280 |
| #5 | "exercise*"[Title/Abstract] OR "physical exercise*"[Title/Abstract] OR "physical activit*"[Title/Abstract] OR "aerobic exercise*"[Title/Abstract] OR "isometric exercise*"[Title/Abstract] OR "activit*"[Title/Abstract] OR "Gymnastics"[Title/Abstract] OR "muscle stretching exercise*"[Title/Abstract] OR "physical conditioning"[Title/Abstract] OR "circuit based exercise*"[Title/Abstract] OR "endurance training"[Title/Abstract] OR "inspiratory training"[Title/Abstract] OR "interval training"[Title/Abstract] OR "continuous training"[Title/Abstract] OR "plyometric exercise*"[Title/Abstract] OR "flexibility exercise"[Title/Abstract] OR "isometric exercise"[Title/Abstract] OR "resistance training"[Title/Abstract] OR "acute exercise*"[Title/Abstract] OR "Jogging"[Title/Abstract] OR "training*"[Title/Abstract] OR "Walking"[Title/Abstract] OR "Cycling"[Title/Abstract] OR "stair climbing"[Title/Abstract] OR "Taichi"[Title/Abstract] OR "Qigong"[Title/Abstract] OR "Swimming"[Title/Abstract] OR "sport*"[Title/Abstract] OR "Running"[Title/Abstract] OR "Yoga"[Title/Abstract] | 4,823,733 |
| #6 | #4 OR #5 | 4,870,743 |
| #7 | "randomized controlled trials as topic"[MeSH Terms] | 177,680 |
| #8 | "randomized controlled trial*"[Title/Abstract] OR "randomized clinical trial*"[Title/Abstract] OR "controlled clinical trial*"[Title/Abstract] OR "double blind method"[Title/Abstract] OR "single blind method"[Title/Abstract] OR "Randomized"[Title/Abstract] OR "control group*"[Title/Abstract] OR "Randomly"[Title/Abstract] OR "random allocation"[Title/Abstract] | 1,542,000 |
| #9 | #7 OR #8 | 1,626,352 |
| #10 | #3 AND #6 AND #9 | 396 |
| **Web of Science (Date: September 12, 2024)** | | |
| No. |  | |
| #1 | (((((((TS=(Prehypertension)) OR TS=(Prehypertension*)) OR TS=(Pre-Hypertension*)) OR TS=(Pre Hypertension*)) OR TS=(Pre-HTN)) OR TS=(PreHTN)) OR TS=(Borderline hypertension*)) OR TS=(Early hypertension*) | 52,892 |
| #2 | ((((((((((((((((((((((((((TS=(Exercise*)) OR TS=(Physical Exercise*)) OR TS=( Physical Activit*)) OR TS=(Aerobic Exercise*)) OR TS=(Isometric Exercise*)) OR TS=(Sport*)) OR TS=(Gymnastics)) OR TS=(Muscle Stretching Exercise*)) OR TS=(Physical Conditioning)) OR TS=(Circuit-Based Exercise*)) OR TS=(Endurance Training)) OR TS=(inspiratory training)) OR TS=(Interval Training)) OR TS=(continuous training)) OR TS=(Plyometric Exercise*)) OR TS=(flexibility exercise)) OR TS=(Isometric exercise)) OR TS=(Resistance Training)) OR TS=(Running)) OR TS=(Jogging)) OR TS=(Swimming)) OR TS=( Walking)) OR TS=(Cycling)) OR TS=(Stair Climbing)) OR TS=( Taichi)) OR TS=(Qigong)) OR TS=(Yoga) | 2,742,358 |
| #3 | (((((TS=(Randomized Controlled Trial*)) OR TS=(Randomized Clinical Trial*)) OR TS=(Controlled Clinical Trial*)) OR TS=(Double-blind method)) OR TS=(Single-blind method)) OR TS=(Random allocation) | 729,059 |
| #4 | #1 AND #2 AND #3 | 628 |
| **Embase (Date: September 12, 2024)** | | |
| No. |  | |
| #1 | 'prehypertension'/exp | 3,912 |
| #2 | prehypertension*:ab,ti OR 'pre hypertension*':ab,ti OR 'pre htn':ab,ti OR prehtn:ab,ti OR 'elevated blood pressure':ab,ti OR 'elevated bp':ab,ti OR 'high normal blood pressure':ab,ti OR 'high normal bp':ab,ti OR 'raised blood pressure':ab,ti OR 'borderline hypertension*':ab,ti OR 'early hypertension*':ab,ti | 21,476 |
| #3 | #1 OR #2 | 22,478 |
| #4 | 'exercise'/exp | 476,282 |
| #5 | 'training'/exp | 129,020 |
| #6 | 'physical activity'/exp | 578,621 |
| #7 | exercise*:ab,ti OR 'exercise capacity':ab,ti OR 'exercise performance':ab,ti OR 'exercise training':ab,ti OR exertion:ab,ti OR 'fitness training':ab,ti OR 'fitness workout':ab,ti OR 'physical conditioning, human':ab,ti OR 'physical effort':ab,ti OR 'physical exercise':ab,ti OR 'physical exertion':ab,ti OR 'physical work-out':ab,ti OR 'physical workout':ab,ti OR 'physical exercise*':ab,ti OR 'physical activit*':ab,ti OR activit*:ab,ti OR 'aerobic exercise*':ab,ti OR 'isometric exercise*':ab,ti OR 'exercise training*':ab,ti OR training:ab,ti OR sport*:ab,ti OR gymnastics:ab,ti OR 'muscle stretching exercise*':ab,ti OR 'circuit training':ab,ti OR 'endurance training':ab,ti OR 'inspiratory training':ab,ti OR 'high-intensity interval training':ab,ti OR 'plyometric exercise*':ab,ti OR 'flexibility exercise':ab,ti OR 'isometric exercise':ab,ti OR 'resistance training':ab,ti OR running:ab,ti OR jogging:ab,ti OR swimming:ab,ti OR walking:ab,ti OR 'stair climbing':ab,ti OR taichi:ab,ti OR qigong:ab,ti OR yoga:ab,ti | 5,992,868 |
| #8 | #4 OR #5 OR #6 OR #7 | 6,328,828 |
| #9 | 'randomized controlled trial'/exp | 842,722 |
| #10 | 'randomized controlled trial*':ab,ti OR 'pragmatic clinical trials as topic':ab,ti OR 'clinical trial*, randomized':ab,ti OR 'trial*, randomized clinical':ab,ti OR 'controlled clinical trial*, randomized':ab,ti OR 'controlled clinical trial':ab,ti OR 'double blind procedure':ab,ti OR 'single blind procedure':ab,ti OR randomly:ab,ti OR randomization:ab,ti OR 'random allocation':ab,ti | 901,751 |
| #11 | #9 OR #10 | 1,402,905 |
| #12 | #3 AND #8 AND #11 | 498 |
| **Cochrane Library (Date: September 12, 2024)** | | |
| No. |  | |
| #1 | MeSH descriptor: [Prehypertension] explode all trees | 262 |
| #2 | (Prehypertension*):ti,ab,kw OR (Pre-Hypertension*):ti,ab,kw OR (Pre Hypertension*):ti,ab,kw OR (Pre-HTN):ti,ab,kw OR (PreHTN):ti,ab,kw (Word variations have been searched) | 6,188 |
| #3 | (Borderline hypertension*):ti,ab,kw OR (Early hypertension*):ti,ab,kw (Word variations have been searched) | 5,918 |
| #4 | #1 OR #2 OR #3 | 11,370 |
| #5 | MeSH descriptor: [Exercise] explode all trees | 39,380 |
| #6 | MeSH descriptor: [Resistance Training] explode all trees | 5,813 |
| #7 | MeSH descriptor: [Endurance Training] explode all trees | 200 |
| #8 | MeSH descriptor: [High-Intensity Interval Training] explode all trees | 1,147 |
| #9 | MeSH descriptor: [Walking] explode all trees | 8,389 |
| #10 | MeSH descriptor: [Jogging] explode all trees | 69 |
| #11 | (Exercise*):ti,ab,kw OR (Aerobic exercise*):ti,ab,kw OR (Physical Conditioning, human):ti,ab,kw OR (Physical activit*):ti,ab,kw OR (Physical Exercise*):ti,ab,kw (Word variations have been searched) | 192,795 |
| #12 | (Circuit-Based Exercise*):ti,ab,kw OR (Inspiratory Training*):ti,ab,kw OR (Exercise Training*):ti,ab,kw OR (Endurance Training*):ti,ab,kw OR (Isometric Exercise*):ti,ab,kw (Word variations have been searched) | 58,145 |
| #13 | (Sport*):ti,ab,kw OR (Gymnastics):ti,ab,kw OR (Muscle Stretching Exercise):ti,ab,kw OR (Walking):ti,ab,kw OR (Swimming):ti,ab,kw (Word variations have been searched) | 63,782 |
| #14 | (High-Intensity Interval Training):ti,ab,kw OR (Plyometric Exercise):ti,ab,kw OR (Flexibility Exercise):ti,ab,kw OR (Resistance Training):ti,ab,kw OR (Stair Climbing):ti,ab,kw (Word variations have been searched) | 29,014 |
| #15 | (Running):ti,ab,kw OR (Jogging):ti,ab,kw OR (Taichi):ti,ab,kw OR (Qigong):ti,ab,kw OR (Yoga):ti,ab,kw (Word variations have been searched) | 35,280 |
| #16 | #5 OR #6 OR #7 OR #8 OR #9 OR #10 OR #11 OR #12 OR #13 OR #14 OR #15 | 244,653 |
| #17 | MeSH descriptor: [Randomized Controlled Trial] explode all trees | 37 |
| #18 | (Randomized Controlled Trial*):ti,ab,kw OR (Clinical Trial*, Randomized):ti,ab,kw OR (Trial*, Randomized Clinical):ti,ab,kw OR (Controlled Clinical Trial*, Randomized):ti,ab,kw OR (Random allocation):ti,ab,kw (Word variations have been searched) | 927,628 |
| #19 | #17 OR #18 | 927,628 |
| #20 | #4 AND #16 AND #19 in Trials (Language: English) | 1,146 |
| **CINAHL ,SPORTDiscus, and Rehabilitation & Sports Medicine Source from EBSCO Interface**  **(Date: September 12, 2024)** | | |
| No. |  | |
| #1 | SU Prehypertension* OR SU Pre-Hypertension* OR SU Pre Hypertension* OR SU Pre-HTN OR SU PreHTN OR SU High normal blood pressure OR SU elevated blood pressure OR SU Borderline hypertension* OR SU Early hypertension* | 3,267 |
| #2 | SU Exercise* OR SU Physical Exercise* OR SU Physical Activit* OR SU Aerobic Exercise* OR SU Isometric Exercise* OR SU Sport* OR SU Gymnastics OR SU Muscle Stretching Exercise* OR SU Physical Conditioning OR SU Circuit-Based Exercise* OR SU Endurance Training OR SU inspiratory training | 1,010,676 |
| #3 | SU Interval Training* OR SU continuous training* OR SU Plyometric Exercise* OR SU flexibility exercise* OR SU Resistance Training* OR SU Running OR SU Jogging OR SU Swimming OR SU Walking OR SU Cycling OR SU Stair Climbing OR SU Taichi | 262,845 |
| #4 | SU Qigong OR SU Yoga | 24,257 |
| #5 | S2 OR S3 OR S4 | 1,185,284 |
| #6 | SU Randomized Controlled Trial* OR SU Randomized Clinical Trial* OR SU Controlled Clinical Trial* OR SU Double-blind method OR SU Single-blind method OR SU Random allocation | 188,685 |
| #7 | S1 AND S5 AND S6 | 30 |

**Supplementary Table 3. Categories of exercise modalities**

| Exercise type | Definition |
| --- | --- |
| AT | Aerobic training is that which uses oxygen in the process of producing energy in the muscles (eg. treadmill or brisk walking, jogging, cycling and swimming etc.) This type of exercise works many muscle groups in a rhythmic manner. It can be performed continuously or in intervals, and at different intensities (low, medium or high) |
| LIT | LIT was consisted of low intensity exercise, whereby 30-50% VO2 peak, or 30-50% heart rate(HR) peak, or equivalent |
| MICT | MICT was defined as sustained aerobic exercise with moderate intensity (50-80% VO2 peak or 50-80% HRpeak, or equivalent) |
| HIIT | HIIT was defined as alternating high-intensity training, whereby ≥80% VO2 peak, or ≥80% heart rate(HR) peak, or equivalent, and were interspersed with short recovery periods by lighter exercise or rest, or equivalent. |
| RT | RT was a training that increases the ability to exert or resist force using load-bearing or resisted exercises including weights, weight-lifting machines or elastic cords |
| IET | Isometric exercise is defined as an exercise in which muscles are sustained contraction against an immovable load or resistance, with no or minimal change in the length of the muscle groups involved(eg.isometric handgrip (IHG), isometric leg extension (ILE), isometric wall squat, and pilates training). |
| Tai chi | Tai chi is a traditional Chinese martial art based on gentle body movements accompanied by relaxation and breathing exercises |
| Yoga | Yoga is exercise with a variety of styles, which typically involve physical poses designed for strengthening and stretching (asanas), controlled breathing (pranayama) and meditation (dhyana) |
| Combined Training | Combination of two types of exercise. |

AT: Aerobic Training; HIIT: High-Intensity Interval Training; IET: Isometric Exercise Training; LIT: Low Intensity Training; MICT: Moderate-Intensity Continuous Training; RT: Resistance Training

**Supplementary Table 4. Excluded full-text with reasons**

| No. | References | Reason for exclusion |
| --- | --- | --- |
| 1 | V Bond 2016^1^ | Not RCTs |
| 2 | J Glodzik 2018^2^ | Not RCTs |
| 3 | K Kukkonen 1982^3^ | Not RCTs |
| 4 | DT Beck 2014^4^ | Unrelated outcome |
| 5 | UG Ogbutor 2022^5^ | Unrelated outcome |
| 6 | T Thida2019^6^ | Unrelated outcome |
| 7 | DT Beck 2013^7^ | Repeated publications |
| 8 | SR Collier 2008^8^ | Repeated publications |
| 9 | C Koppelstätter 2024^9^ | Repeated publications |
| 10 | P Prasertsri 2010^10^ | Repeated publications |
| 11 | FG Márquez-Celedonio 2009^11^ | foreign language |
| 12 | K Chan 2020^12^ | conference abstracts |
| 13 | S Hunter 2019^13^ | conference abstracts |
| 14 | X Li 2020^14^ | conference abstracts |
| 15 | Q Peng 2020^15^ | conference abstracts |
| 16 | T Ramkumar 2014^16^ | conference abstracts |
| 17 | J Wiles 2022^17^ | conference abstracts |
| 18 | M Filip 2015^18^ | conference abstracts |
| 19 | S Tantiprasoplap 2020^19^ | academic thesis |
| 20 | AM Afify 2023^20^ | Study on acute blood pressure assessment |
| 21 | DMP Karoline 2015^21^ | Study on acute blood pressure assessment |
| 22 | N Mevada 2024^22^ | Study on acute blood pressure assessment |
| 23 | S Park 2006^23^ | Study on acute blood pressure assessment |
| 24 | S Rodrigues 2021^24^ | Study on acute blood pressure assessment |
| 25 | Y Saxena 2016^25^ | Study on acute blood pressure assessment |
| 26 | ZS Zeigler 2016^26^ | Study on acute blood pressure assessment |
| 27 | ZS Zeigler 2015^27^ | Study on acute blood pressure assessment |
| 28 | S Chen 2016^28^ | Ineligible interventions |
| 29 | JW Hughes 2013^29^ | Ineligible interventions |
| 30 | M Jalilian 2024^30^ | Ineligible interventions |
| 31 | J Kalinowski 2021^31^ | Ineligible interventions |
| 32 | PA Modesti 2015^32^ | Ineligible interventions |
| 33 | S Pengpid 2019^33^ | Ineligible interventions |
| 34 | ER Serber 2016^34^ | Ineligible interventions |
| 35 | SZ Wang 2010^35^ | Ineligible interventions |
| 36 | RBR- Fdkw 2018^36^ | Type of disease not met |
| 37 | A Aminuddin 2011^37^ | Type of disease not met |
| 38 | E An 2019^38^ | Type of disease not met |
| 39 | K Anjana 2022^39^ | Type of disease not met |
| 40 | BJ Arsenault 2009^40^ | Type of disease not met |
| 41 | GI Ash 2017^41^ | Type of disease not met |
| 42 | Y Bai 2022^42^ | Type of disease not met |
| 43 | BB Barone 2009^43^ | Type of disease not met |
| 44 | JA Blumenthal 2010^44^ | Type of disease not met |
| 45 | JA Blumenthal 2000^45^ | Type of disease not met |
| 46 | FP Boeno 2020^46^ | Type of disease not met |
| 47 | TS Church 2007^47^ | Type of disease not met |
| 48 | DL Cohen 2011^48^ | Type of disease not met |
| 49 | SR Collier 2011^49^ | Type of disease not met |
| 50 | SR Collier 2009^50^ | Type of disease not met |
| 51 | LA Crist 2012^51^ | Type of disease not met |
| 52 | P Delgado-Floody 2020^52^ | Type of disease not met |
| 53 | KM Edwards 2011^53^ | Type of disease not met |
| 54 | A Figueroa 2014^54^ | Type of disease not met |
| 55 | J Gabiola 2020^55^ | Type of disease not met |
| 56 | M Hagins 2014^56^ | Type of disease not met |
| 57 | KS Heffernan 2013^57^ | Type of disease not met |
| 58 | J Lea 2024^58^ | Type of disease not met |
| 59 | M Miyashita 2011^59^ | Type of disease not met |
| 60 | GE Moore 2006^60^ | Type of disease not met |
| 61 | NCT 2009^61^ | Type of disease not met |
| 62 | JE Park 2011^62^ | Type of disease not met |
| 63 | JE Park 2017^63^ | Type of disease not met |
| 64 | J Park 2012^64^ | Type of disease not met |
| 65 | D Pinto 2022^65^ | Type of disease not met |
| 66 | S Punia 2019^66^ | Type of disease not met |
| 67 | S Punia 2022^67^ | Type of disease not met |
| 68 | BA Staffileno 2007^68^ | Type of disease not met |
| 69 | R Supriya 2018^69^ | Type of disease not met |
| 70 | E Tekn 2023^70^ | Type of disease not met |
| 71 | JAG Wijnen 1994^71^ | Type of disease not met |
| 72 | A Wong 2016^72^ | Type of disease not met |
| 73 | SK Xu 2019^73^ | Type of disease not met |
| 74 | K Yuenyongchaiwat 2024^74^ | Type of disease not met |
| 75 | RBR- Cgp H 2018^75^ | Type of disease not met |
| 76 | S Sehgal 2023^76^ | Incomplete data |
| 77 | CTRI 2024^77^ | Study protocol |
| 78 | ChiCTR 2019^78^ | Study protocol |
| 79 | NCT 2022^79^ | Study protocol |
| 80 | NCT 2012^80^ | Study protocol |
| 81 | NCT 2021^81^ | Study protocol |
| 82 | TCTR 2017^82^ | Study protocol |

**References**

1 V Bond, BH Curry, RG Adams, et al. Cardiovascular Responses to an Isometric Handgrip Exercise in Females with Prehypertension. *N Am J Med Sci* 2016;8(6):243-49.

2 J Glodzik, K Rewiuk, J Adamiak, et al. Controlled aerobic training improves endothelial function and modifies vascular remodeling in healthy adults with high normal blood pressure. *J Physiol Pharmacol* 2018;69(5).

3 K Kukkonen, R Rauramaa, E Voutilainen, E Lansimies. Physical training of middle-aged men with borderline hypertension. *Annals of Clinical Research* 1982;14(Suppl. 34):139-45.

4 DT Beck, JS Martin, DP Casey, RW Braith. Exercise training improves endothelial function in resistance arteries of young prehypertensives. *J Hum Hypertens* 2014;28(5):303-09.

5 UG Ogbutor, EK Nwangwa, BC Nwogueze, et al. Proinflammatory and Anti-inflammatory Cytokine Response to Isometric Handgrip Exercise and the Effects of Duration and Intensity of the Isometric Efforts in Prehypertensive Participants. *J Chiropr Med* 2022;21(3):177-86.

6 T Thida, A Yupin, P Sunida. Effect of Self-Management Exercise Program on Physical Fitness among People with Prehypertension and Obesity: A Quasi Experiment Study. *Pac Rim Int J Nurs Res Thail* 2019;23(1):6-17.

7 DT Beck, JS Martin, DP Casey, RW Braith. Exercise training reduces peripheral arterial stiffness and myocardial oxygen demand in young prehypertensive subjects. *Am J Hypertens* 2013;26(9):1093-102.

8 SR Collier, JA Kanaley, R Carhart, et al. Effect of 4 weeks of aerobic or resistance exercise training on arterial stiffness, blood flow and blood pressure in pre- and stage-1 hypertensives. 2008;22(10):678-86.

9 C Koppelstätter. Effect of Tai Chi vs aerobic exercise on blood pressure in patients with prehypertension. *J Hyperton* 2024;28(1):28-29.

10 P Prasertsri, S Singsanan, C Chonanant, O Boonla, P Trongtosak. Effects of arm swing exercise training on cardiac autonomic modulation, cardiovascular risk factors, and electrolytes in persons aged 60–80 years with prehypertension: A randomized controlled trial. *J Exerc Sci Fit* 2010;17(2):47-54.

11 FG Márquez-Celedonio, O Téxon-Fernández, A Chávez-Negrete, et al. Clinical Effect of Lifestyle Modification on Cardiovascular Risk in Prehypertensives: PREHIPER I Study. *Rev Esp Cardiol* 2009;62(1):86-90.

12 K Chan, E Wee, M Mok, H Ler. Effects of resistance band training and body weight training on selected health parameters of pre-hypertensive adults. 2020;21(SUPPL 1).

13 S Hunter, AT Fadeyi, J Shadiow. Hot yoga and hypertension: Exploration of a novel lifestyle intervention. *Hypertension* 2019;74.

14 X Li, Q Peng, K Tan, G Li. Influence of Taiji exercise on blood pressure and mood state of people with borderline hypertension. 2020;126:81.

15 Q Peng, X Li, G Li. Influence of different intensity aerobic exercise on blood pressure and mental health of people with borderline hypertension. 2020;126:79-80.

16 T Ramkumar, S Senthil Kumar, P Pravati, et al. Addition of yoga therapy to standard lifestyle modification improve cardiovascular autonomic function and metabolic derangement in prehypertensive subjects: A randomized controlled study. *Eur J Prev Cardiol* 2014;21(1):S111.

17 J Wiles, J Lea, J O'Driscoll. A feasibility study examining the prescription of isometric exercise training for blood pressure management using rating of perceived exertion. *J Hum Hypertens* 2022;36:13.

18 M Filip, J Glodzik, T Mikolajczyk, et al. Modulatory effect of physical excercise on immune system in prehypertensive patients-a randomized cross-over study. *Atherosclerosis* 2015;241(1):e187.

19 S Tantiprasoplap, N Piaseu, V Kanungsukkasem, S Taneepanichskul. A randomized controlled trial comparing the effects of an arm swing exercise and low sodium intake education program with low sodium intake education alone on cardiovascular outcomes in postmenopausal women with prehypertension. 2020;103(1):22-31.

20 AM Afify. Effect of Diaphragmatic Breathing Exercise on Cardiovascular Parameters Following Noise Exposure in Pre Hypertensive Adults. 2023;30(7):e79-86.

21 DMP Karoline, MM Sales, DAJ Alves, et al. Effects of aerobic exercise intensity on 24-h ambulatory blood pressure in individuals with type 2 diabetes and prehypertension. *J Phys Ther Sci* 2015;27(1):51-56.

22 N Mevada, M Mehta, F Oza, J Chokshi. Immediate Effects of Calf Muscle Release via Foam Rolling and Active Stretching on Blood Pressure, Heart Rate and the Rate Pressure Product in People with High-Normal Blood Pressure – A Comparative Study. *Cardiovasc Innov Appl* 2024;9.

23 S Park, LD Rink, JP Wallace. Accumulation of physical activity leads to a greater blood pressure reduction than a single continuous session, in prehypertension. *J Hypertens* 2006;24(9):1761-70.

24 S Rodrigues, R Verardino, M Costa, et al. Evaluation of hemodynamic and vascular responses after a continuous exercise session of moderate intensity and high intensity intervals in individuals with normal and high normal blood pressure. *Artery Res* 2021;27:S8.

25 Y Saxena, R Gupta, A Moinuddin, R Narwal. Blood pressure reduction following accumulated physical activity in prehypertensive. *J Family Med Prim Care* 2016;5(2):349-56.

26 ZS Zeigler, PD Swan. Acute effects of whole-body vibration with resistance exercise on postexercise blood pressure and oxygen consumption in prehypertensive adults. *J Exerc Sci Fit* 2016;14(1):14-23.

27 ZS Zeigler, PD Swan, DM Bhammar, GA Gaesser. Walking Workstation Use Reduces Ambulatory Blood Pressure in Adults With Prehypertension. *J Phys Act Health* 2015;12:S119-27.

28 S Chen, P Sun, S Wang, G Lin, T Wang. Effects of heart rate variability biofeedback on cardiovascular responses and autonomic sympathovagal modulation following stressor tasks in prehypertensives. *J Hum Hypertens* 2016;30(2):105-11.

29 JW Hughes, DM Fresco, R Myerscough, et al. Randomized controlled trial of mindfulness-based stress reduction for prehypertension. *Psychosom Med* 2013;75(8):721-28.

30 M Jalilian, M Koushki, M Saeedfiroozabadi, et al. The effect of a theory-based health education program on physical activity and blood pressure in middle-aged women with pre-hypertension: A cluster randomized trial. *J Hum Behav Soc Environ* 2024;34(7):1075-88.

31 J Kalinowski, SF Castaneda, MA Allison, et al. Telephone-based mindfulness training in diverse prehypertensive women: Results of a pilot randomized controlled trial. *Circulation* 2021;143(SUPPL 1).

32 PA Modesti, A Ferrari, C Bazzini, M Boddi. Time sequence of autonomic changes induced by daily slow-breathing sessions. *Clin Auton Res* 2015;25(2):95-104.

33 S Pengpid, K Peltzer, I Jayasvasti, et al. Two-year results of a community-based randomized controlled lifestyle intervention trial to control prehypertension and/or prediabetes in Thailand: a brief report. *Int J Gen Med* 2019;12:131-35.

34 ER Serber, J Ciccolo, K Palmer, et al. The feasibility of exercise videogames for cardiovascular risk reduction among adults: a pilot for "Wii heart fitness". *J Sports Med Phys Fitness* 2016;56(3):319-27.

35 SZ Wang, S Li, XY Xu, et al. Effect of slow abdominal breathing combined with biofeedback on blood pressure and heart rate variability in prehypertension. *J Altern Complement Med* 2010;16(10):1039-45.

36 RBR- Fdkw. Effect of high intensity interval training on physical capacity, body composition, quality of life and autonomic cardiac control of hypertensions. 2018.

37 A Aminuddin, Z Zakaria, NAMM Nordin, et al. Effect of graded aerobic exercise training on blood pressure changes in women with elevated blood pressure. *International Medical Journal* 2011;18(3):207-11.

38 E An, ML Brecht, LV Doering, et al. Mindfulness and lifestyle medicine lowers blood pressure in hypertension. *Circulation* 2019;140.

39 K Anjana, R Archana, JK Mukkadan. Effect of om chanting and yoga nidra on blood pressure and lipid profile in hypertension - A randomized controlled trial. *J Ayurveda Integr Med* 2022;13(4):100657.

40 BJ Arsenault, M Côté, A Cartier, et al. Effect of exercise training on cardiometabolic risk markers among sedentary, but metabolically healthy overweight or obese post-menopausal women with elevated blood pressure. *Atherosclerosis* 2009;207(2):530-33.

41 GI Ash, BA Taylor, PD Thompson, et al. The antihypertensive effects of aerobic versus isometric handgrip resistance exercise. *J Hypertens* 2017;35(2):291-99.

42 Y Bai, R Burns, N Gell, W Byun. A randomized trial to promote physical activity in adult pre-hypertensive and hypertensive patients. *J Sports Sci* 2022;40(14):1648-57.

43 BB Barone, NY Wang, AC Bacher, KJ Stewart. Decreased exercise blood pressure in older adults after exercise training: contributions of increased fitness and decreased fatness. *Br J Sports Med* 2009;43(1):52-56.

44 JA Blumenthal, MA Babyak, A Hinderliter, et al. Effects of the DASH diet alone and in combination with exercise and weight loss on blood pressure and cardiovascular biomarkers in men and women with high blood pressure: The ENCORE study. *Archives of Internal Medicine* 2010;170(2):126-35.

45 JA Blumenthal, A Sherwood, ECD Gullette, et al. Exercise and weight loss reduce blood pressure in men and women with mild hypertension: Effects on cardiovascular, metabolic, and hemodynamic functioning. *Archives of Internal Medicine* 2000;160(13):1947-58.

46 FP Boeno, TR Ramis, SV Munhoz, et al. Effect of aerobic and resistance exercise training on inflammation, endothelial function and ambulatory blood pressure in middle-aged hypertensive patients. *J Hypertens* 2020;38(12):2501-09.

47 TS Church, CP Earnest, JS Skinner, SN Blair. Effects of different doses of physical activity oncardiorespiratory fitness among sedentary, overweight or obese postmenopausal women with elevated blood pressure: A randomized controlled trial. *Jama* 2007;297(19):2081-91.

48 DL Cohen, LT Bloedon, RL Rothman, et al. Iyengar yoga versus enhanced usual care on blood pressure in patients with prehypertension to stage i hypertension: A randomized controlled trial. *Evid Based Complement Alternat Med* 2011;2011.

49 SR Collier, V Frechette, K Sandberg, et al. Sex differences in resting hemodynamics and arterial stiffness following 4 weeks of resistance versus aerobic exercise training in individuals with pre-hypertension to stage 1 hypertension. *Biol Sex Differ* 2011;2(1):9.

50 SR Collier, JA Kanaley, R Carhart Jr., et al. Cardiac autonomic function and baroreflex changes following 4 weeks of resistance versus aerobic training in individuals with pre-hypertension. *Acta Physiol (Oxf)* 2009;195(3):339-48.

51 LA Crist, CM Champagne, L Corsino, et al. Influence of change in aerobic fitness and weight on prevalence of metabolic syndrome. *Prev Chronic Dis* 2012;9:E68.

52 P Delgado-Floody, M Izquierdo, R Ramírez-Vélez, et al. Effect of High-Intensity Interval Training on Body Composition, Cardiorespiratory Fitness, Blood Pressure, and Substrate Utilization During Exercise Among Prehypertensive and Hypertensive Patients With Excessive Adiposity. *Front Physiol* 2020;11:558910.

53 KM Edwards, KL Wilson, J Sadja, MG Ziegler, PJ Mills. Effects on blood pressure and autonomic nervous system function of a 12-week exercise or exercise plus DASH-diet intervention in individuals with elevated blood pressure. *Acta Physiol (Oxf)* 2011;203(3):343-50.

54 A Figueroa, R Kalfon, TA Madzima, A Wong. Whole-body vibration exercise training reduces arterial stiffness in postmenopausal women with prehypertension and hypertension. *Menopause* 2014;21(2):131-36.

55 J Gabiola, D Morales, O Quizon, et al. The EffectiveNess of LIfestyle with Diet and Physical Activity Education ProGram Among Prehypertensives and Stage 1 HyperTENsives in an Urban Community Setting (ENLIGHTEN) Study. *J Community Health* 2020;45(3):478-87.

56 M Hagins, A Rundle, NS Consedine, SBS Khalsa. A Randomized Controlled Trial Comparing the Effects of Yoga With an Active Control on Ambulatory Blood Pressure in Individuals With Prehypertension and Stage 1 Hypertension. *J Clin Hypertens (Greenwich)* 2014;16(1):54-62.

57 KS Heffernan, ES Yoon, JE Sharman, et al. Resistance exercise training reduces arterial reservoir pressure in older adults with prehypertension and hypertension. *Hypertens Res* 2013;36(5):422-27.

58 J Lea, JM O'Driscoll, JD Wiles. The implementation of a home-based isometric wall squat intervention using ratings of perceived exertion to select and control exercise intensity: a pilot study in normotensive and pre-hypertensive adults. *Eur J Appl Physiol* 2024;124(1):281-93.

59 M Miyashita, SF Burns, DJ Stensel. Accumulating short bouts of running reduces resting blood pressure in young normotensive/pre-hypertensive men. *J Sports Sci* 2011;29(14):1473-82.

60 GE Moore, MJ LaMonte. Can exercise lower blood pressure in mildly hypertensive older persons? Commentary. 2006;16(5):451-52.

61 NCT. Lifestyle Modification and Blood Pressure Study. 2009.

62 JE Park, S Hong, M Lee, et al. Randomized Controlled Trial of Qigong for Treatment of Prehypertension and Mild Essential Hypertension. 2011.

63 JE Park, JE Kim, S Jung, et al. The Effect of Dongeui Qigong for Prehypertension and Mild Essential Hypertension. *Evid Based Complement Alternat Med* 2017;2017.

64 J Park, S Hong, T Park, et al. A randomized controlled trial for the use of qigong in the treatment of pre and mild essential hypertension. 2012;12.

65 D Pinto, ND Rato, C Garcia, et al. EFFECTS OF HOME-BASED ISOMETRIC HANDGRIP OR AEROBIC EXERCISE TRAINING IN OLDER ADULTS WITH PREHYPERTENSION AND HYPERTENSION. *J Hypertens* 2022;40:e263.

66 S Punia, S Kulandaivelan. Effect of 8 weeks body-weight resistance training on high-normal blood pressure and stage 1 hypertension subjects-pilot study to validate the protocol. *Indian Journal of Public Health Research and Development* 2019;10(2):175-80.

67 S Punia, V Singh, S Joshi, M Malik, M Saini. Effects of walking in individuals with prehypertension and stage 1 hypertension in India: a randomised controlled trial. *International Journal of Therapy & Rehabilitation* 2022;29(4):1-10.

68 BA Staffileno, A Minnick, LA Coke, SM Hollenberg. Blood pressure responses to lifestyle physical activity among young, hypertension-prone African-American women. *The Journal of cardiovascular nursing* 2007;22(2):107-17.

69 R Supriya, AP Yu, PH Lee, et al. Yoga training modulates adipokines in adults with high-normal blood pressure and metabolic syndrome. *Scand J Med Sci Sports* 2018;28(3):1130-38.

70 E Tekn, F Ünver, YT Yaylalı. Investigation of the effects of 8-week Nordic and traditional walking training on blood pressure in prehypertensive postmenopausal women. *Spor Hekimligi Dergisi/Turkish Journal of Sports Medicine* 2023;58(3):112-17.

71 JAG Wijnen, MJF Kool, MA Van Baak, et al. Effect of exercise training on ambulatory blood pressure. *Int J Sports Med* 1994;15(1):10-15.

72 A Wong, S Alvarez-Alvarado, AW Kinsey, A Figueroa. Whole-Body Vibration Exercise Therapy Improves Cardiac Autonomic Function and Blood Pressure in Obese Pre- and Stage 1 Hypertensive Postmenopausal Women. 2016;22(12):970-76.

73 SK Xu, Y Chen, CY Liu, et al. A randomized cross-over study on the blood pressure lowering effect of the combined passive head-up and -down movement with Device-Guided slow breathing. 2019;28(5):291-99.

74 K Yuenyongchaiwat, K Changsri, S Harnmanop, et al. Effects of slow breathing training on hemodynamic changes, cardiac autonomic function and neuroendocrine response in people with high blood pressure: A randomized control trial. *Journal of Bodywork & Movement Therapies* 2024;37:136-41.

75 RBR- Cgp H. Effect of aerobic training combined with strength training with elastic tube on functional capacity, blood pressure and muscular strength in hypertensive patients. 2018.

76 S Sehgal, J Daniel, M Sharma. A Comparative Study to Find the Effect of Aerobic Exercise Training Versus Resistance Exercise Training in Adults with Pre-Hypertension. *Indian Journal of Public Health Research and Development* 2023;14(1):347-52.

77 CTRI. Comparative effect of Myofascual release technique and lifestyle modification in prehypertension individuals A RANDOMIZED CONTROLLED TRIAL. 2024.

78 ChiCTR. A Randomized Controlled Trial on Effect of Tai Chi versus aerobic exercise on Blood Pressure in Prehypertension Patients. 2019.

79 NCT. Stretching vs Walking for Lowering Blood Pressure. 2022.

80 NCT. Running Against Prehypertension Trial (RAPT): a Pilot Trial. 2012.

81 NCT. Inspiratory Muscle Strength Training for Lowering Blood Pressure and Improving Endothelial Function in Postmenopausal Women: comparison With "Standard of Care" Aerobic Exercise. 2021.

82 TCTR. A Randomized Controlled Trial Comparing the Effects of Arm-swing Exercise and Low Sodium Intake Education Program with Low Sodium Intake Education Alone on Blood pressure, Heart rate and Cardiorespiratory fitness in Postmenopausal women with Prehypertension. 2017.

**Supplementary Table 5. Meta-regression analysis results**

| Covariate | Mean | SD | 2.5% | 25% | 50% | 75% | 97.5% |
| --- | --- | --- | --- | --- | --- | --- | --- |
| SBP | | | | |  |  |  |
| year | 0.8044 | 8.420 | -13.814 | -5.0785 | 0.1525 | 6.6126 | 17.5692 |
| Age | 0.8160 | 6.158 | -11.349 | -3.1181 | 0.6423 | 4.87569 | 12.54246 |
| Female ratio | 0.2535 | 6.092 | -11.666 | -3.678 | 0.1927 | 4.0795 | 12.6162 |
| Duration | 2.9399 | 4.006 | -5.6515 | 0.5271 | 2.7317 | 5.105 | 12.8725 |
| Frequency | 10.2804 | 17.653 | -16.319 | -0.2284 | 5.0104 | 21.5716 | 47.0923 |
| DBP | | | | |  |  |  |
| year | 10.3841 | 11.513 | -9.609 | 1.2549 | 10.8982 | 19.65283 | 33.8871 |
| Age | 4.8070 | 4.0771 | -2.980 | 2.0484 | 4.6834 | 7.329 | 13.6710 |
| Female ratio | 3.6761 | 5.434 | -6.683 | 0.1561 | 3.5517 | 7.0461 | 14.89300 |
| Duration | 6.7802 | 10.462 | -9.049 | 0.8268 | 5.2591 | 12.2010 | 27.976 |
| Frequency | 27.3118 | 39.928 | -10.274 | -1.0061 | 7.4733 | 43.7895 | 109.0319 |

**Supplementary Table 6. CINeMA confidence rating**

CINeMA results for SBP

| Comparison | Number of Studies | Within-study bias | Reporting bias | Indirectness | Imprecision | Heterogeneity | Incoherence | Confidence rating | Reason(s) for downgrading |
| --- | --- | --- | --- | --- | --- | --- | --- | --- | --- |
| Mixed evidence | | | | | | | | |  |
| ATRT vs MICT | 1 | No concerns | Low risk | No concerns | Some concerns | Some concerns | No concerns | Low | \| Imprecision \| Heterogeneity \| |
| ATRT vs RT | 1 | No concerns | Low risk | No concerns | Some concerns | Some concerns | Major concerns | Very low | \| Imprecision \| Heterogeneity \| Incoherence \| |
| ATRT vs control | 2 | No concerns | Low risk | No concerns | No concerns | Some concerns | No concerns | Moderate | \| Heterogeneity \| |
| HIIT vs MICT | 1 | Some concerns | Low risk | No concerns | Major concerns | No concerns | No concerns | Very low | \| Within-study bias \| Imprecision \| |
| HIIT vs control | 1 | Some concerns | Low risk | No concerns | Some concerns | No concerns | No concerns | Low | \| Within-study bias \| Imprecision \| |
| IET vs control | 3 | No concerns | Low risk | No concerns | No concerns | Some concerns | No concerns | Moderate | \| Heterogeneity \| |
| LIT vs Yoga | 1 | Some concerns | Low risk | No concerns | No concerns | Some concerns | No concerns | Low | \| Within-study bias \| Heterogeneity \| |
| LIT vs control | 1 | Some concerns | Low risk | No concerns | Major concerns | No concerns | No concerns | Very low | \| Within-study bias \| Imprecision \| |
| MICT vs RT | 2 | No concerns | Low risk | No concerns | Some concerns | Some concerns | No concerns | Low | \| Imprecision \| Heterogeneity \| |
| MICT vs Tai chi | 1 | No concerns | Low risk | No concerns | Major concerns | No concerns | No concerns | Low | \| Imprecision \| |
| MICT vs control | 6 | Some concerns | Low risk | No concerns | No concerns | Some concerns | No concerns | Low | \| Within-study bias \| Heterogeneity \| |
| RT vs control | 3 | Some concerns | Low risk | No concerns | No concerns | Some concerns | No concerns | Low | \| Within-study bias \| Heterogeneity \| |
| Yoga vs control | 3 | Some concerns | Low risk | No concerns | No concerns | Some concerns | No concerns | Low | \| Within-study bias \| Heterogeneity \| |
| Indirect evidence | | | | | | | | |  |
| ATRT vs HIIT | -- | Some concerns | Low risk | No concerns | Major concerns | No concerns | No concerns | Very low | \| Within-study bias \| Imprecision \| |
| ATRT vs IET | -- | No concerns | Low risk | No concerns | Major concerns | No concerns | No concerns | Very low | \| Imprecision \| |
| ATRT vs LIT | -- | Some concerns | Low risk | No concerns | No concerns | Some concerns | No concerns | Low | \| Within-study bias \| Heterogeneity \| |
| ATRT vs Tai chi | -- | No concerns | Low risk | No concerns | Major concerns | No concerns | No concerns | Low | \| Imprecision \| |
| ATRT vs Yoga | -- | Some concerns | Low risk | No concerns | Some concerns | Some concerns | No concerns | Very low | \| Within-study bias \| Imprecision \| Heterogeneity \| |
| HIIT vs IET | -- | Some concerns | Low risk | No concerns | Major concerns | No concerns | No concerns | Very low | \| Within-study bias \| Imprecision \| |
| HIIT vs LIT | -- | Some concerns | Low risk | No concerns | Some concerns | Some concerns | No concerns | Very low | \| Within-study bias \| Imprecision \| Heterogeneity \| |
| HIIT vs RT | -- | Some concerns | Low risk | No concerns | Major concerns | No concerns | No concerns | Very low | \| Within-study bias \| Imprecision \| |
| HIIT vs Tai chi | -- | Some concerns | Low risk | No concerns | Major concerns | No concerns | No concerns | Very low | \| Within-study bias \| Imprecision \| |
| HIIT vs Yoga | -- | Some concerns | Low risk | No concerns | Major concerns | No concerns | No concerns | Very low | \| Within-study bias \| Imprecision \| |
| IET vs LIT | -- | Some concerns | Low risk | No concerns | No concerns | Some concerns | No concerns | Low | \| Within-study bias \| Heterogeneity \| |
| IET vs MICT | -- | No concerns | Low risk | No concerns | Some concerns | Some concerns | No concerns | Low | \| Imprecision \| Heterogeneity \| |
| IET vs RT | -- | No concerns | Low risk | No concerns | Some concerns | Some concerns | No concerns | Low | \| Imprecision \| Heterogeneity \| |
| IET vs Tai chi | -- | No concerns | Low risk | No concerns | Major concerns | No concerns | No concerns | Low | \| Imprecision \| |
| IET vs Yoga | -- | Some concerns | Low risk | No concerns | Some concerns | Some concerns | No concerns | Very low | \| Within-study bias \| Imprecision \| Heterogeneity \| |
| LIT vs MICT | -- | Some concerns | Low risk | No concerns | No concerns | Some concerns | No concerns | Low | \| Within-study bias \| Heterogeneity \| |
| LIT vs RT | -- | Some concerns | Low risk | No concerns | Some concerns | Some concerns | No concerns | Very low | \| Within-study bias \| Imprecision \| Heterogeneity \| |
| LIT vs Tai chi | -- | Some concerns | Low risk | No concerns | Some concerns | No concerns | No concerns | Low | \| Within-study bias \| Imprecision \| |
| MICT vs Yoga | -- | Some concerns | Low risk | No concerns | Some concerns | Some concerns | No concerns | Very low | \| Within-study bias \| Imprecision \| Heterogeneity \| |
| RT vs Tai chi | -- | No concerns | Low risk | No concerns | Some concerns | Some concerns | No concerns | Low | \| Imprecision \| Heterogeneity \| |
| RT vs Yoga | -- | Some concerns | Low risk | No concerns | Some concerns | Some concerns | No concerns | Very low | \| Within-study bias \| Imprecision \| Heterogeneity \| |
| Tai chi vs Yoga | -- | No concerns | Low risk | No concerns | Major concerns | No concerns | No concerns | Low | \| Imprecision \| |
| Tai chi vs control | -- | No concerns | Low risk | No concerns | No concerns | Some concerns | No concerns | Moderate | \| Heterogeneity \| |

CINeMA results for DBP

| Comparison | Number of Studies | Within-study bias | Reporting bias | Indirectness | Imprecision | Heterogeneity | Incoherence | Confidence rating | Reason(s) for downgrading |
| --- | --- | --- | --- | --- | --- | --- | --- | --- | --- |
| Mixed evidence | | | | | | | | |  |
| ATRT vs MICT | 1 | No concerns | Low risk | No concerns | Some concerns | Some concerns | No concerns | Low | \| Imprecision \| Heterogeneity \| |
| ATRT vs RT | 1 | No concerns | Low risk | No concerns | Major concerns | No concerns | No concerns | Low | \| Imprecision \| |
| ATRT vs control | 2 | No concerns | Low risk | No concerns | Some concerns | Some concerns | No concerns | Low | \| Imprecision \| Heterogeneity \| |
| HIIT vs MICT | 1 | Some concerns | Low risk | No concerns | Major concerns | No concerns | No concerns | Very low | \| Within-study bias \| Imprecision \| |
| HIIT vs control | 1 | Some concerns | Low risk | No concerns | Some concerns | Some concerns | No concerns | Very low | \| Within-study bias \| Imprecision \| Heterogeneity \| |
| IET vs control | 3 | No concerns | Low risk | No concerns | No concerns | Some concerns | No concerns | Moderate | \| Heterogeneity \| |
| LIT vs Yoga | 1 | Some concerns | Low risk | No concerns | No concerns | Some concerns | No concerns | Low | \| Within-study bias \| Heterogeneity \| |
| LIT vs control | 1 | Some concerns | Low risk | No concerns | Some concerns | Some concerns | No concerns | Very low | \| Within-study bias \| Imprecision \| Heterogeneity \| |
| MICT vs RT | 2 | No concerns | Low risk | No concerns | Some concerns | Some concerns | No concerns | Low | \| Imprecision \| Heterogeneity \| |
| MICT vs Tai chi | 1 | No concerns | Low risk | No concerns | Major concerns | No concerns | No concerns | Low | \| Imprecision \| |
| MICT vs control | 6 | Some concerns | Low risk | No concerns | No concerns | Some concerns | No concerns | Low | \| Within-study bias \| Heterogeneity \| |
| RT vs control | 3 | Some concerns | Low risk | No concerns | Some concerns | Some concerns | No concerns | Very low | \| Within-study bias \| Imprecision \| Heterogeneity \| |
| Yoga vs control | 3 | Some concerns | Low risk | No concerns | No concerns | Some concerns | No concerns | Low | \| Within-study bias \| Heterogeneity \| |
| Indirect evidence | | | | | | | | |  |
| ATRT vs HIIT | -- | Some concerns | Low risk | No concerns | Major concerns | No concerns | No concerns | Very low | \| Within-study bias \| Imprecision \| |
| ATRT vs IET | -- | No concerns | Low risk | No concerns | Some concerns | No concerns | No concerns | Moderate | \| Imprecision \| |
| ATRT vs LIT | -- | Some concerns | Low risk | No concerns | Some concerns | Some concerns | No concerns | Very low | \| Within-study bias \| Imprecision \| Heterogeneity \| |
| ATRT vs Tai chi | -- | No concerns | Low risk | No concerns | Major concerns | No concerns | No concerns | Low | \| Imprecision \| |
| ATRT vs Yoga | -- | Some concerns | Low risk | No concerns | Some concerns | Some concerns | No concerns | Very low | \| Within-study bias \| Imprecision \| Heterogeneity \| |
| HIIT vs IET | -- | Some concerns | Low risk | No concerns | Some concerns | Some concerns | No concerns | Very low | \| Within-study bias \| Imprecision \| Heterogeneity \| |
| HIIT vs LIT | -- | Some concerns | Low risk | No concerns | Some concerns | Some concerns | No concerns | Very low | \| Within-study bias \| Imprecision \| Heterogeneity \| |
| HIIT vs RT | -- | Some concerns | Low risk | No concerns | Major concerns | No concerns | No concerns | Very low | \| Within-study bias \| Imprecision \| |
| HIIT vs Tai chi | -- | Some concerns | Low risk | No concerns | Major concerns | No concerns | No concerns | Very low | \| Within-study bias \| Imprecision \| |
| HIIT vs Yoga | -- | Some concerns | Low risk | No concerns | Major concerns | No concerns | No concerns | Very low | \| Within-study bias \| Imprecision \| |
| IET vs LIT | -- | Some concerns | Low risk | No concerns | No concerns | Some concerns | No concerns | Low | \| Within-study bias \| Heterogeneity \| |
| IET vs MICT | -- | No concerns | Low risk | No concerns | Some concerns | Some concerns | No concerns | Low | \| Imprecision \| Heterogeneity \| |
| IET vs RT | -- | No concerns | Low risk | No concerns | Some concerns | Some concerns | No concerns | Low | \| Imprecision \| Heterogeneity \| |
| IET vs Tai chi | -- | No concerns | Low risk | No concerns | Major concerns | No concerns | No concerns | Low | \| Imprecision \| |
| IET vs Yoga | -- | Some concerns | Low risk | No concerns | Some concerns | Some concerns | No concerns | Very low | \| Within-study bias \| Imprecision \| Heterogeneity \| |
| LIT vs MICT | -- | Some concerns | Low risk | No concerns | No concerns | Some concerns | No concerns | Low | \| Within-study bias \| Heterogeneity \| |
| LIT vs RT | -- | Some concerns | Low risk | No concerns | Some concerns | Some concerns | No concerns | Very low | \| Within-study bias \| Imprecision \| Heterogeneity \| |
| LIT vs Tai chi | -- | Some concerns | Low risk | No concerns | Some concerns | Some concerns | No concerns | Very low | \| Within-study bias \| Imprecision \| Heterogeneity \| |
| MICT vs Yoga | -- | Some concerns | Low risk | No concerns | Some concerns | Some concerns | No concerns | Very low | \| Within-study bias \| Imprecision \| Heterogeneity \| |
| RT vs Tai chi | -- | No concerns | Low risk | No concerns | Major concerns | No concerns | No concerns | Low | \| Imprecision \| |
| RT vs Yoga | -- | Some concerns | Low risk | No concerns | Some concerns | Some concerns | No concerns | Very low | \| Within-study bias \| Imprecision \| Heterogeneity \| |
| Tai chi vs Yoga | -- | No concerns | Low risk | No concerns | Major concerns | No concerns | No concerns | Low | \| Imprecision \| |
| Tai chi vs control | -- | No concerns | Low risk | No concerns | Some concerns | Some concerns | No concerns | Low | \| Imprecision \| Heterogeneity \| |

CINeMA results for FMD

| Comparison | Number of Studies | Within-study bias | Reporting bias | Indirectness | Imprecision | Heterogeneity | Incoherence | Confidence rating | Reason(s) for downgrading |
| --- | --- | --- | --- | --- | --- | --- | --- | --- | --- |
| Mixed evidence | | | | | | | | |  |
| ATRT vs Control | 1 | Major concerns | Low risk | No concerns | Major concerns | No concerns | Major concerns | Very low | \| Within-study bias \| Imprecision \| Incoherence \| |
| Control vs MICT | 3 | Some concerns | Low risk | No concerns | No concerns | Major concerns | No concerns | Very low | \| Within-study bias \| Heterogeneity \| |
| Control vs RT | 2 | Some concerns | Low risk | No concerns | Major concerns | No concerns | Major concerns | Very low | \| Within-study bias \| Imprecision \| Incoherence \| |
| MICT vs RT | 1 | Some concerns | Low risk | No concerns | Some concerns | Some concerns | No concerns | Very low | \| Within-study bias \| Imprecision \| Heterogeneity \| |
| Indirect evidence | | | | | | | | |  |
| ATRT vs MICT | -- | Some concerns | Low risk | No concerns | Major concerns | No concerns | Major concerns | Very low | \| Within-study bias \| Imprecision \| Incoherence \| |
| ATRT vs RT | -- | Some concerns | Low risk | No concerns | Major concerns | No concerns | Major concerns | Very low | \| Within-study bias \| Imprecision \| Incoherence \| |

CINeMA results for PWV

| Comparison | Number of Studies | Within-study bias | Reporting bias | Indirectness | Imprecision | Heterogeneity | Incoherence | Confidence rating | Reason(s) for downgrading |
| --- | --- | --- | --- | --- | --- | --- | --- | --- | --- |
| Mixed evidence | | | | | | | | |  |
| ATRT vs Control | 2 | Some concerns | Low risk | No concerns | Some concerns | Some concerns | No concerns | Very low | \| Within-study bias \| Imprecision \| Heterogeneity \| |
| Control vs IET | 1 | No concerns | Low risk | No concerns | Some concerns | No concerns | No concerns | Moderate | \| Imprecision \| |
| Control vs MICT | 1 | Some concerns | Low risk | No concerns | Some concerns | No concerns | No concerns | Low | \| Within-study bias \| Imprecision \| |
| Control vs RT | 1 | Some concerns | Low risk | No concerns | No concerns | No concerns | No concerns | Moderate | \| Within-study bias \| |
| Indirect evidence | | | | | | | | |  |
| ATRT vs IET | -- | No concerns | Low risk | No concerns | Some concerns | No concerns | No concerns | Moderate | \| Imprecision \| |
| ATRT vs MICT | -- | Some concerns | Low risk | No concerns | Some concerns | No concerns | No concerns | Low | \| Within-study bias \| Imprecision \| |
| ATRT vs RT | -- | Some concerns | Low risk | No concerns | Some concerns | No concerns | No concerns | Low | \| Within-study bias \| Imprecision \| |
| IET vs MICT | -- | No concerns | Low risk | No concerns | Some concerns | No concerns | No concerns | Moderate | \| Imprecision \| |
| IET vs RT | -- | No concerns | Low risk | No concerns | Some concerns | No concerns | No concerns | Moderate | \| Imprecision \| |
| MICT vs RT | -- | Some concerns | Low risk | No concerns | Some concerns | No concerns | No concerns | Low | \| Within-study bias \| Imprecision \| |

**Supplementary Figure 1. Detailed information of the risk of bias assessment for each eligible study**


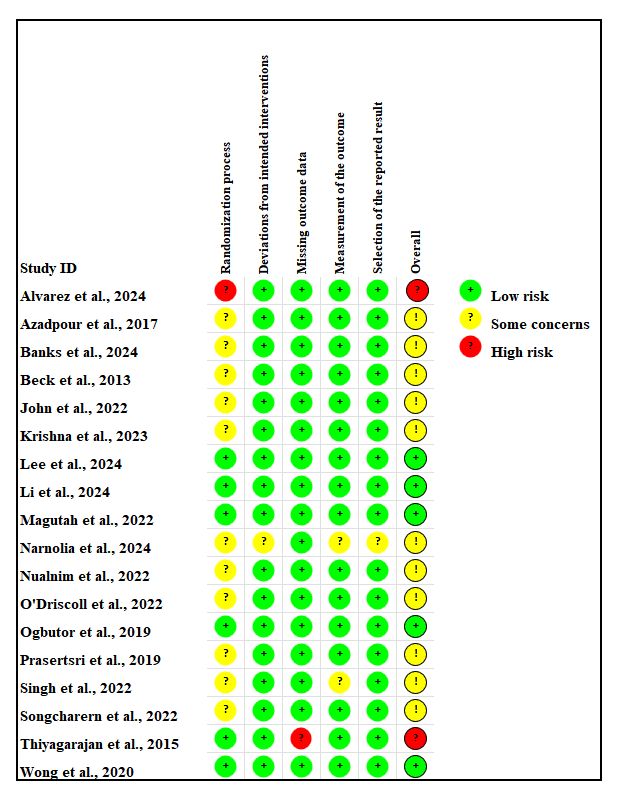


**Supplementary Figure 2. Forest plot of each modality of exercise compared to Control**


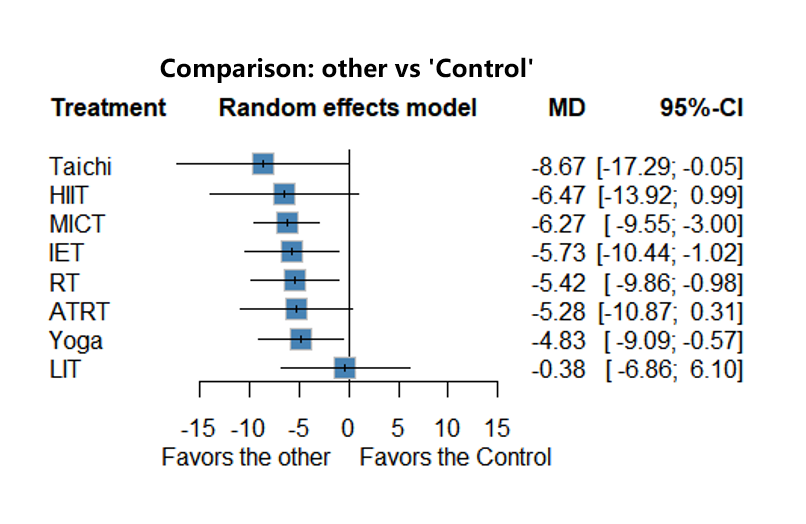


**Supplementary Figure 2.1. Forest plot compared to Control for SBP**


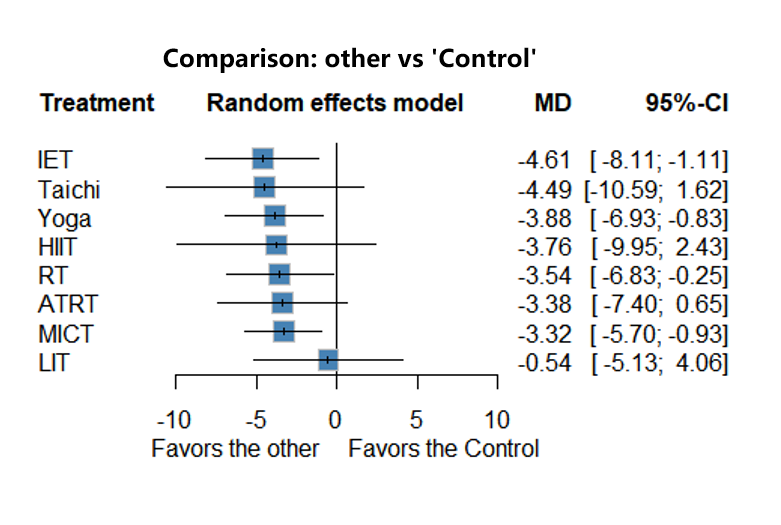


**Supplementary Figure 2.2. Forest plot compared to Control for DBP**


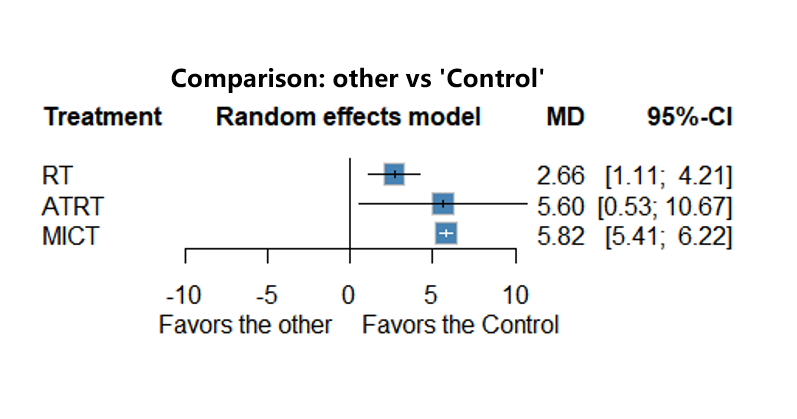


**Supplementary Figure 2.3. Forest plot compared to Control for FMD**


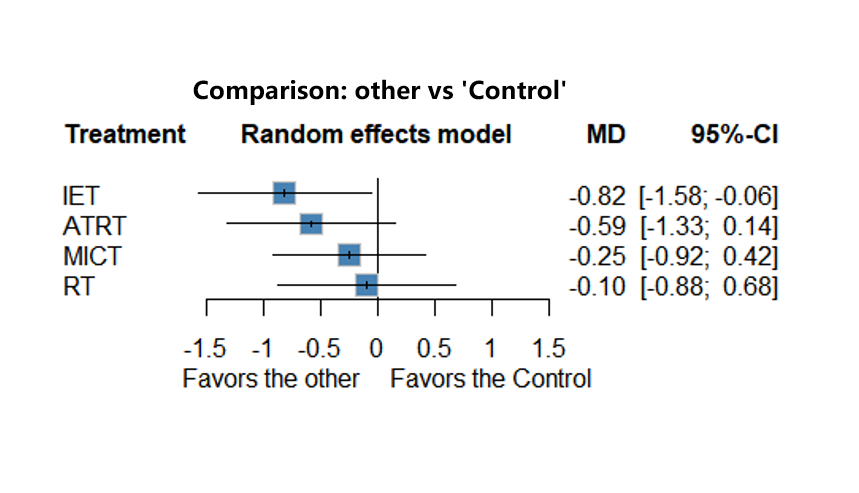


**Supplementary Figure 2.4. Forest plot compared to Control for PWV**

**Supplementary Figure 3. League heat plot for SBP**

**
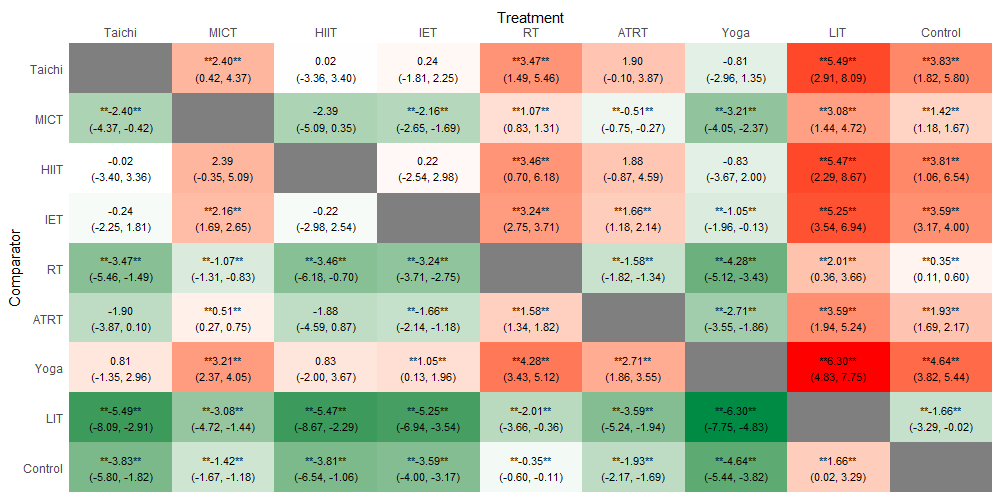
**

**Supplementary Figure 4. League heat plot for DBP**

**
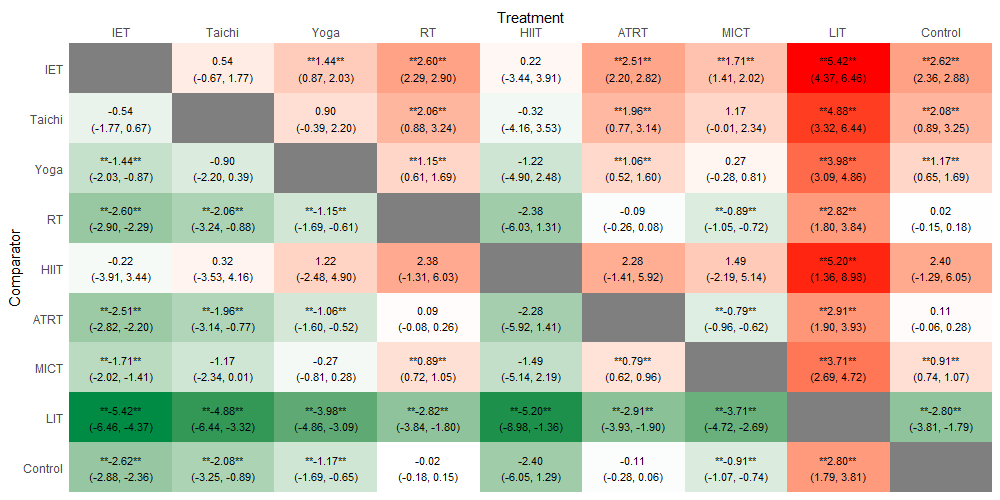
**

**Supplementary Figure 5. Ranking superiority and NMA estimates for FMD**


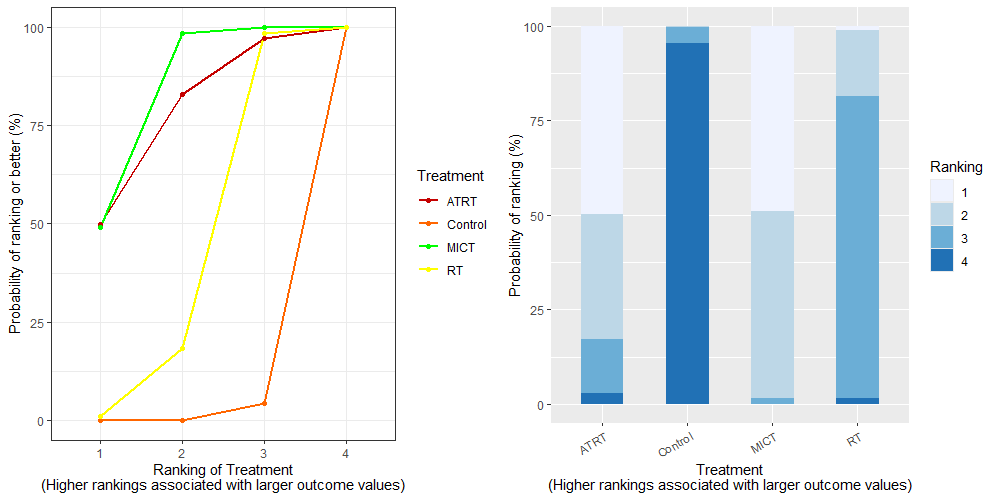


**Supplementary Figure 5.1. Treatment rank probabilities plot for FMD**


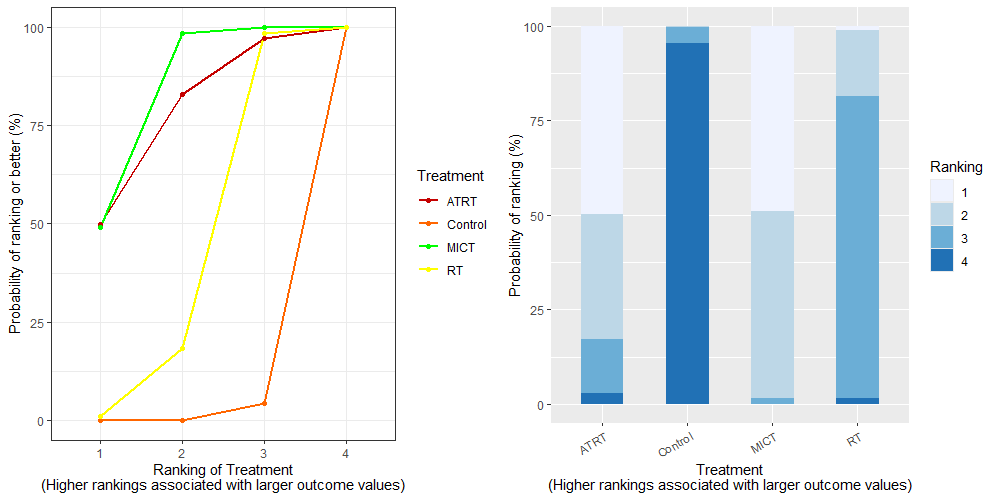


**Supplementary Figure 5.2. SUCRA plot for FMD**


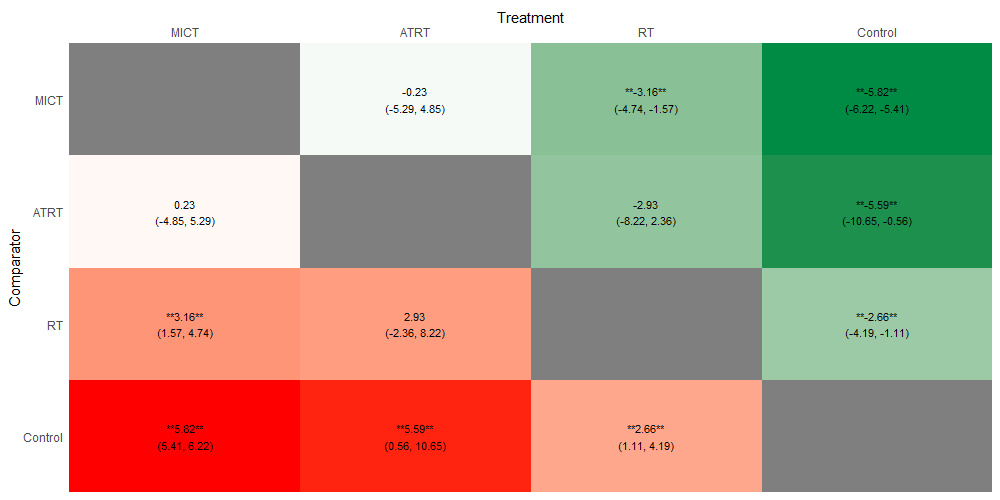


**Supplementary Figure 5.3. League heat plot for FMD**

**Supplementary Figure 6. Ranking superiority and NMA estimates for PWV**

**
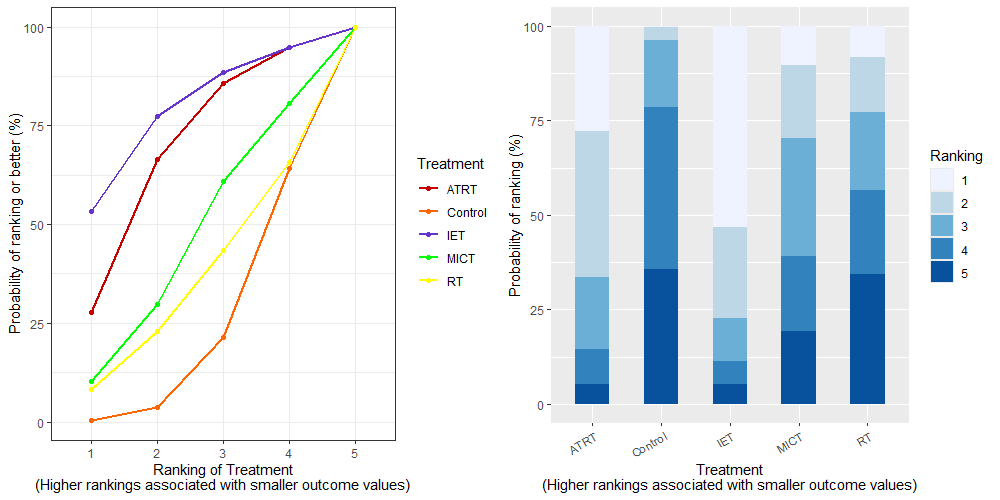
**

**Supplementary Figure 6.1. Treatment rank probabilities plot for PWV**

**
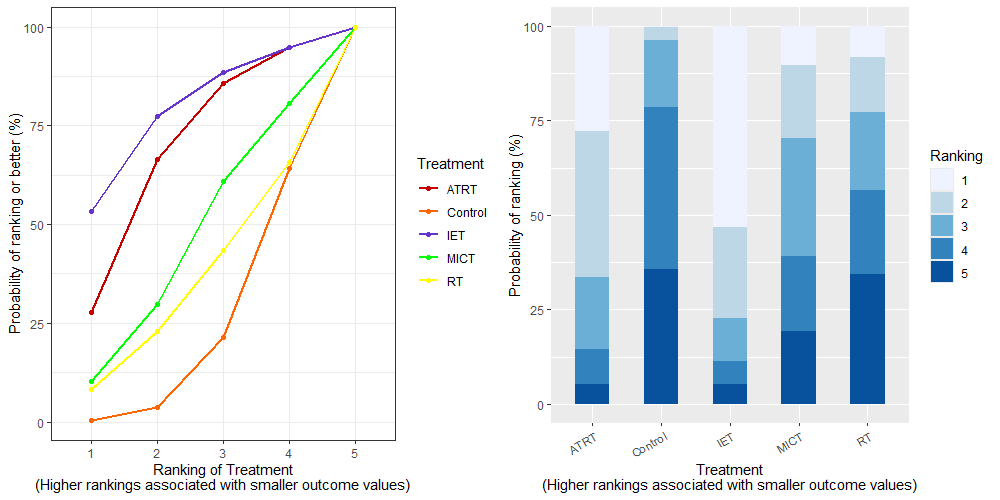
**

**Supplementary Figure 6.2. SUCRA plot for PWV**

**
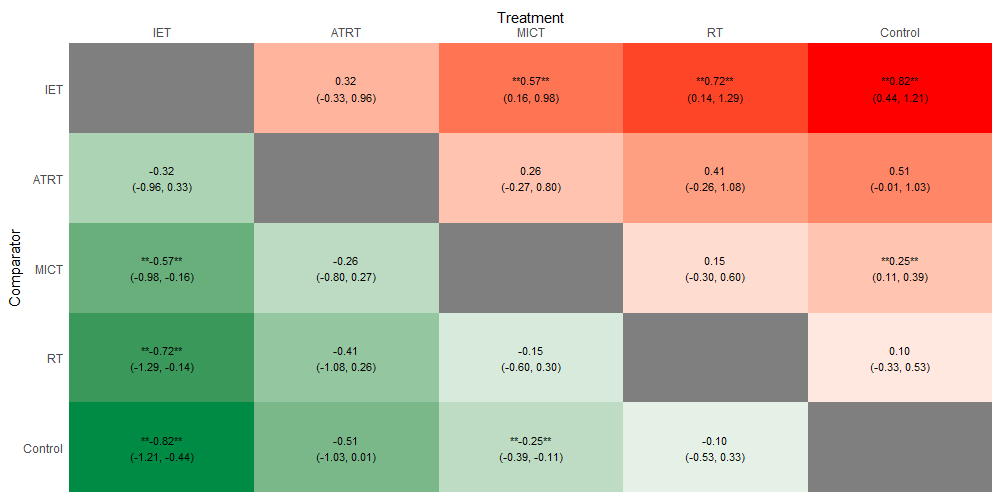
**

**Supplementary Figure 6.3. League heat plot for PWV**

**Supplementary Figure 7. Fixed and random effects modeling**


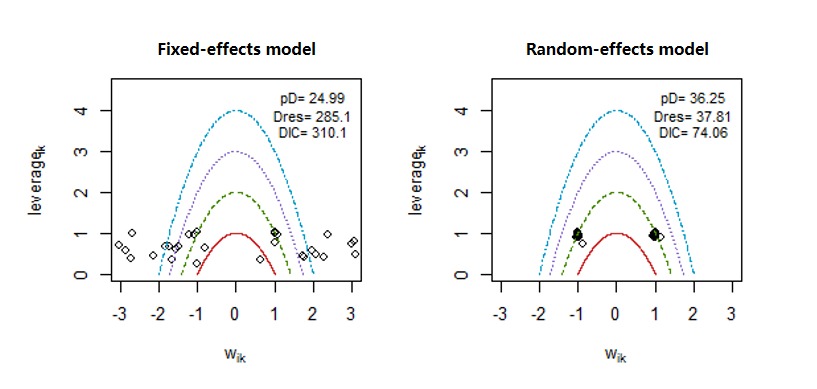


**Supplementary Figure 7.1. Leverage plots and DIC for fixed vs. random effects modeling in SBP**


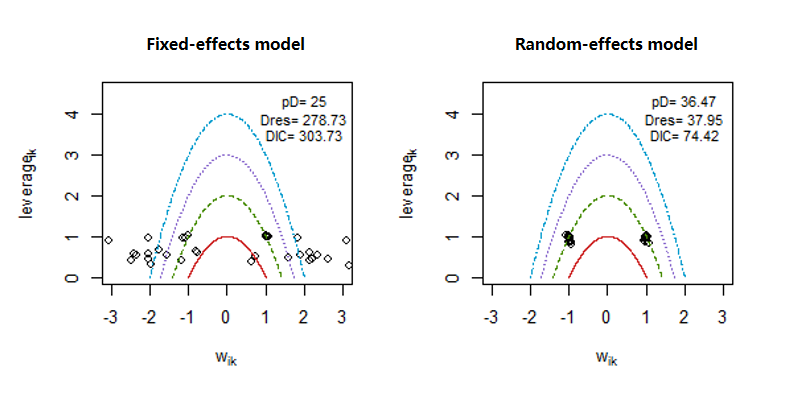


**Supplementary Figure 7.2. Leverage plots and DIC for fixed vs. random effects modeling in DBP**


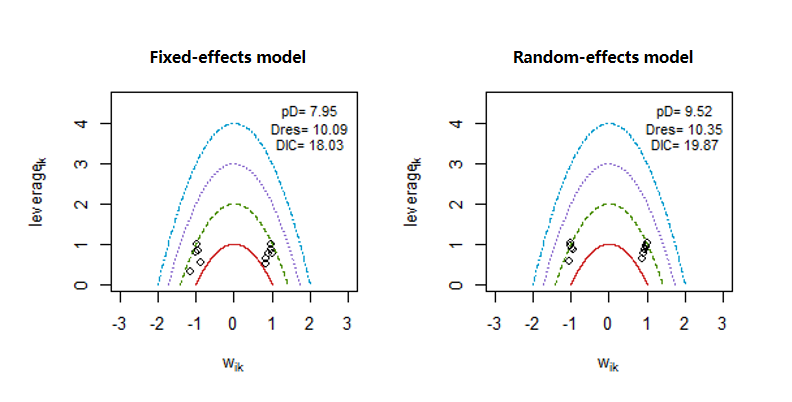


**Supplementary Figure 7.3. Leverage plots and DIC for fixed vs. random effects modeling in FMD**


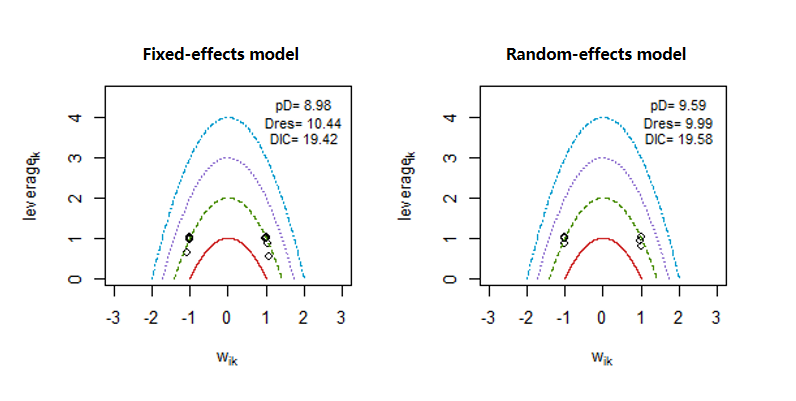


**Supplementary Figure 7.4. Leverage plots and DIC for fixed vs. random effects modeling in PWV**

**Supplementary Figure 8. Node-splitting analysis**


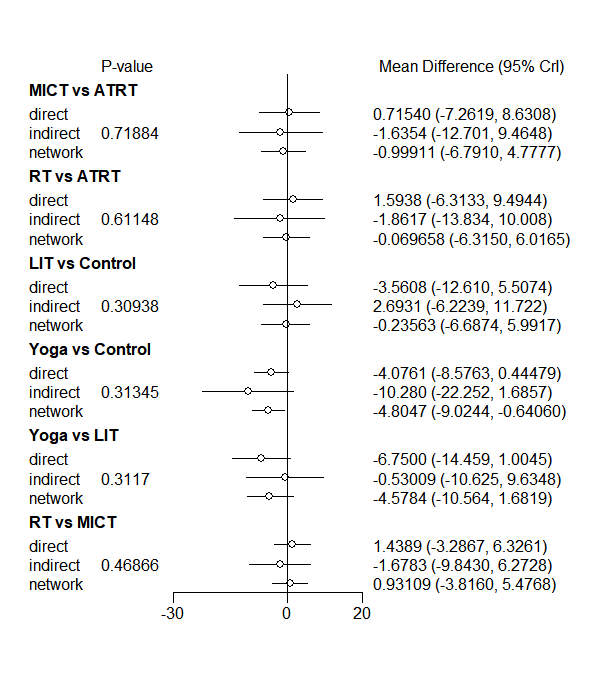


**Supplementary Figure 8.1. Node-splitting analysis comparing the mean differences and 95% CrI between direct, indirect and network meta-analysis evidence for SBP**


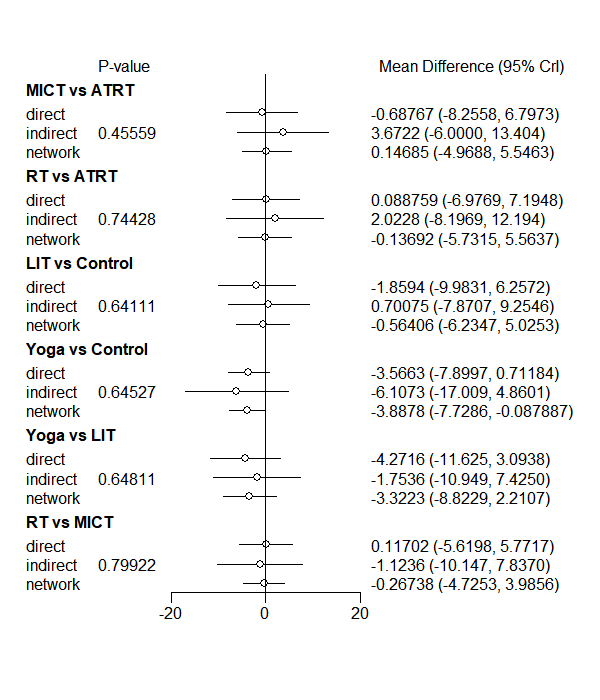


**Supplementary Figure 8.2. Node-splitting analysis comparing the mean differences and 95% CrI between direct, indirect and network meta-analysis evidence for DBP**

**Supplementary Figure 9. Sensitivity analysis of the primary outcomes**


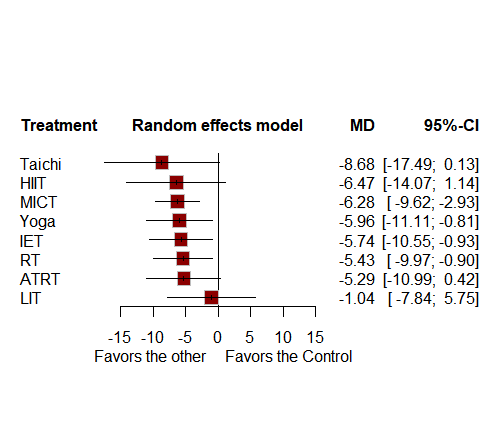


**Supplementary Figure 9.1. Forest plot of the pooled SBP effect of each modality of exercise compared to the control group without high risk of bias studies**


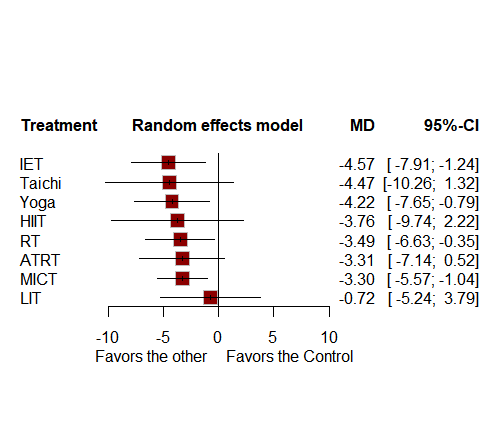


**Supplementary Figure 9.2. Forest plot of the pooled DBP effect of each modality of exercise compared to the control group without high risk of bias studies**

**Supplementary Figure 10. Plot of funnel**


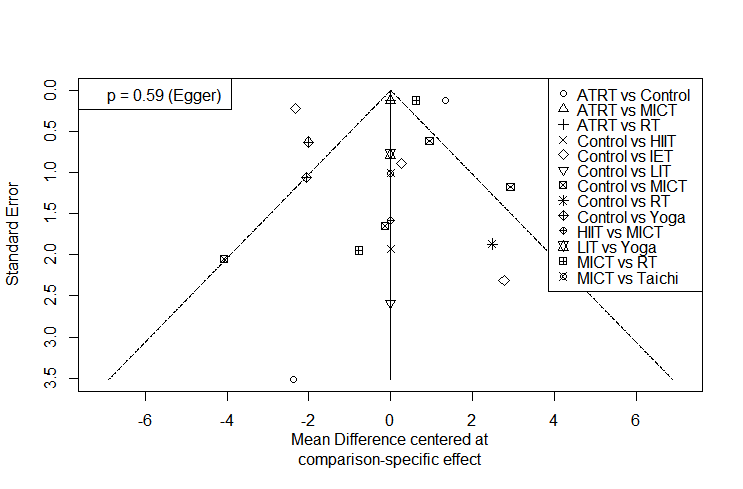


**Supplementary Figure 10.1. Funnel plots for studies reporting SBP**

**
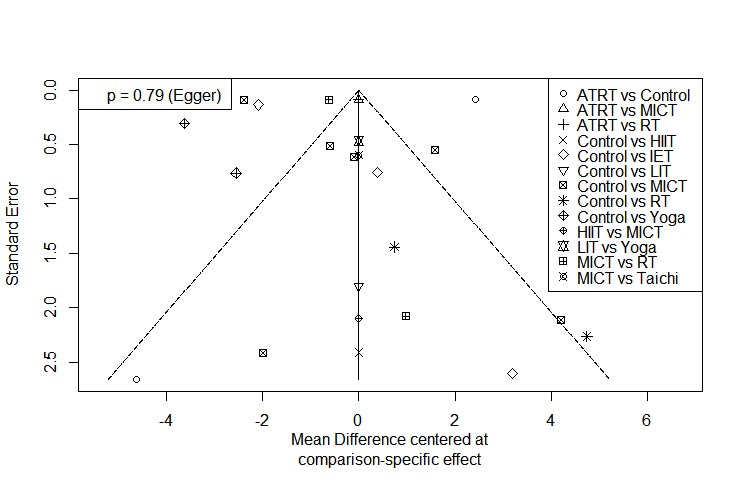
**

**Supplementary Figure 10.2. Funnel plots for studies reporting DBP**
